# Healthy lifestyle choices: new insights into vitiligo management

**DOI:** 10.3389/fimmu.2024.1440705

**Published:** 2024-11-18

**Authors:** Xin Liang, Fei Guo, Qian Fan, Xiaoce Cai, Jiao Wang, Jiale Chen, Fang Liu, Yuhua Du, Yan Chen, Xin Li

**Affiliations:** ^1^ Chinese Medicine Department, Songnan Town Community Health Service Center, Shanghai, China; ^2^ Department of Dermatology, Yueyang Hospital of Integrated Traditional Chinese and Western Medicine, Shanghai University of Traditional Chinese Medicine, Shanghai, China; ^3^ Institute of Dermatology, Shanghai Academy of Traditional Chinese Medicine, Shanghai, China

**Keywords:** lifestyle, systematic review, vitiligo, diet, exercise

## Abstract

**Background:**

The treatment of vitiligo is complex, and providing guidance based on lifestyle habits is a good option that has not been summarized or analyzed.

**Objective:**

To elucidate the relationship between vitiligo and lifestyle factors.

**Methods:**

Four databases (PubMed, Embase, Cochrane, and China National Knowledge Internet) were searched for articles published between 1980 and December 2022. Keywords such as smoking, drinking, exercise, diet, and sleep were used.

**Results:**

Based on the search strategy, 875 relevant studies were retrieved, and 73 were included in this study, of which 41 studies with 8,542 patients with vitiligo were included in the meta-analysis. Vitamin C [mean difference (MD), −0.342; 95% confidence interval (CI), −1.090–0.407; p >0.05), folic acid (MD, −1.463; 95% CI, −7.133–4.208; p >0.05), and selenium (MD, 0.350; 95% CI, −0.687–1.387; p >0.05) levels did not differ between the groups. Vitamin E (MD, −1.408; 95% CI, −2.611–−0.206; p <0.05), vitamin B12 (MD, −0.951; 95% CI, −1.672–−0.275; p <0.05), copper (MD, −0.719; 95% CI, −1.185–−0.252, p <0.005), and zinc (MD, −0.642; 95% CI, −0.731–−0.554; p <0.001) levels were lower in the vitiligo group than in the control group. The serum iron level of the vitiligo group was significantly higher than that of the control group (MD, 1.181; 95% CI, 0.390–1.972; p <0.005). Finally, more participants in the vitiligo group smoked and drank alcohol than those in the control group.

**Limitations:**

Most studies are from Eastern countries; thus, extrapolating these results to Western populations is questionable. The significant heterogeneity may be attributed to the different stages, types, duration, center settings, population registries, etc., which seriously impair the validity of the results.

**Conclusions:**

Patients with vitiligo should reduce smoking and alcohol consumption and take appropriate vitamin E, B12, copper, and zinc supplements. However, vitamin C, vitamin D, selenium, iron, and folic acid supplements are unnecessary. Moreover, they should consider sun protection and avoid permanent hair dye use. Patients with vitiligo may experience sleep disturbances and sexual dysfunction, and these patients should seek help from a specialist if necessary.

**Systematic review registration:**

https://www.crd.york.ac.uk/prospero/#recordDetails, identifier CRD42023480757.

## Introduction

Vitiligo is an autoimmune skin disease associated with features such as chronic loss of melanocyte function and number and the formation of white patches or spots on the skin that impact one’s esthetic appearance. The disease affects approximately 0.1%–2% of people worldwide and profoundly impact patients’ quality of life.

Treatment options for vitiligo remain limited (1), as its pathogenesis remains unclear and may be related to oxidative stress, genetic, and environmental factors. Researchers have classified this disease as an autoimmune disease (2–5). Vitiligo is currently treated with narrow-band ultraviolet (UV) B-rays (UVB), 308-nm excimer lasers, calcium-regulated phosphatase inhibitors, glucocorticoids, Janus kinase inhibitors, surgical treatments, cosmetic covers, and others (6, 7). Owing to the limited treatment options for vitiligo, adopting a lifestyle approach to manage vitiligo symptoms and progression may be necessary. Although many studies have reported the influence of various lifestyle factors on vitiligo, no comprehensive literature review currently summarizes these factors to guide patients in their lifestyle choices. Therefore, in this study, we extensively reviewed the literature to summarize these findings for the first time. Our aim was to provide valuable information on vitiligo treatment and empower patients with better insights into managing their condition.

In this systematic review, we analyzed smoking, alcohol consumption, diet, exercise, light exposure, height, sleep, and permanent hair dye use data to provide more targeted, effective, and safe life coaching for patients suffering from this disfiguring disease. The summary of these studies may serve as a valuable resource for guiding future research in the field of vitiligo.

## Materials and methods

We performed a systematic review and meta-analysis to assess the association between vitiligo and lifestyle. This study was conducted according to the Meta-analysis of Observational Studies in Epidemiology (MOOSE) guidelines and registered with PROSPERO (CRD42023480757), an international registry of prospective systematic evaluations https://www.crd.york.ac.uk/PROSPERO/(**Supplementary Tables S1**, **S2**).

### Data sources and searches

To explore the relationship between lifestyle and vitiligo, three reviewers (Xin Liang, Fei Guo, and Xin Li) systematically searched relevant publications from the EMBASE, PubMed, and Cochrane library electronic databases and the China National Knowledge Internet using the following keywords: “vitiligo,” “sports,” ‘‘smoking,’’ “alcohol consumption,” “insomnia,” “diet,” “vitamin C,” “vitamin D,” “vitamin E,” “vitamin B12,” “vitamin A,” “folic acid,” “zinc,” “copper,” “selenium,” “iron,” and “sunshine.” Our comprehensive search encompassed articles written in English, spanning from January 1980 to December 2022. Additionally, the references of the retrieved articles were manually scanned.

### Inclusion and exclusion criteria

**Figure 1:**
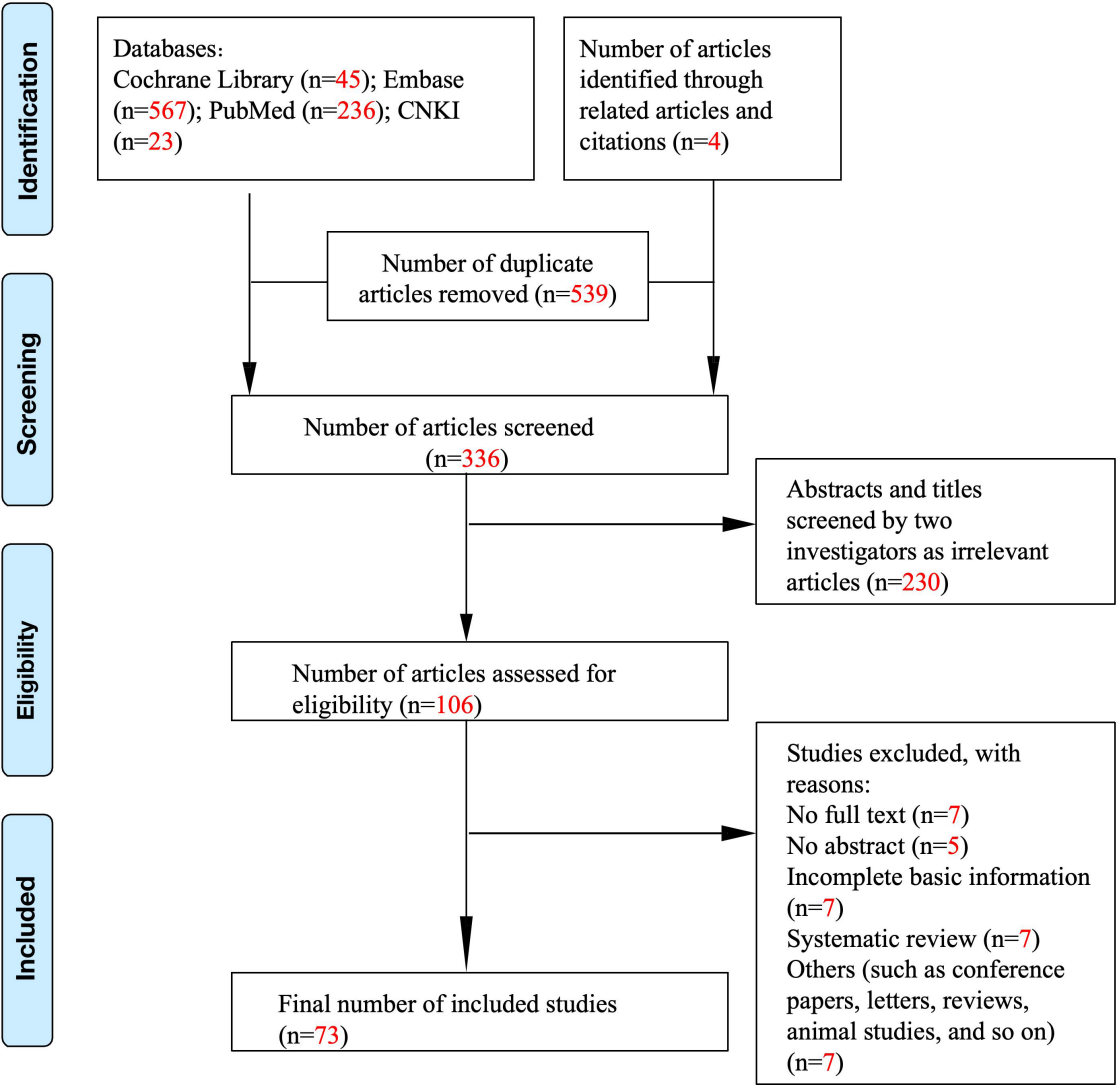
Literature search and study selection process.

Studies were selected based on the following criteria: (1) randomized controlled trials and observational studies, (2) human studies only, (3) studies describing the relationship between vitiligo and lifestyle habits, and (4) studies assessing the impact of lifestyle habits on the course of vitiligo. The exclusion criteria were as follows: (1) animal studies and (2) inability to contact the corresponding author for data. Initially, 706 publications were included (**Figure 1**). After a manual review of the reference lists of the included studies, three additional articles were identified. Then, these studies were carefully reviewed. Finally, 73 studies were included in this article. **Figure 1** shows a flowchart of the screening process.

### Data extraction and quality assessment

Three reviewers, including the first author, independently checked the data within each selected study against a predetermined data extraction form, encompassing study, participant, and outcome characteristics. The Newcastle–Ottawa scale (8) was used to assess the study quality.

### Data synthesis and analysis

All analyses were performed using Stata software. The weighted mean difference/standardized mean difference and corresponding 95% confidence intervals (CIs) were aggregated to assess the association between serum vitamin E, C, zinc, copper, B12, and folic acid levels and vitiligo. Heterogeneity was tested using the I^2^ statistic, with I^2^ >50% considered highly heterogeneous. A random-effects model was employed owing to the observed heterogeneity, and Egger’s test was used to assess publication bias. Finally, a sensitivity analysis was performed to explore the impact of potential sources of heterogeneity (**Supplementary Figures S1**-**S4**).

## Results

### Search results

A total of 875 articles were retrieved from PubMed, Cochrane Library, Embase databases, and China National Knowledge Infrastructure (CNKI) (**Figure 1**). In total, 539 duplicate items were excluded from further assessment. After screening the abstracts and titles, 106 studies remained. After a comprehensive review of the selected articles, 33 studies were excluded, and 73 studies were included in this systematic review, of which 41 studies with a total of 8,542 patients with vitiligo were finally included in the meta-analysis (**Tables 1**, **2**).

**Table 1:** Characteristics of the included studies and the Newcastle–Ottawa Scale (NOS) Quality Assessment Table.

Authors (publication year, country/region)	Studies	Sample size	Intervention	Study design	Duration	Main results	NOS
Juhlin et al. (1997, Sweden) (9)	Improvement of vitiligo after oral treatment with vitamin B12 and folic acid and the importance of sun exposure	Patients with vitiligo, 100	Test: tablets containing vitamin B12 (1 mg cyanocobalamin) and folic acid (5 mg) should be taken twice daily for 3 months. Goal: to expose their skin to the sun in summer and UVB irradiation in winter	Single-arm study	NR	Repigmentation was clearly noted in 52 patients; 37 had been exposed to sunlight from April to September in Sweden, and six had been exposed to UVB lamps once or twice weekly in the winter. Repigmentation was most evident in sun-exposed areas, where 38% of the patients had previously noted repigmentation during the summer months. Total repigmentation was observed in six patients. The spread of vitiligo stopped in 64% of the patients after treatment.	2
Lajevardi et al. (2015, Providence) (10)	Vitiligo and associated pigmentation, sun exposure, and lifestyle factors in women	63,315 women	Pigmentation, sun exposure, and lifestyle factors	Cohort study	12 years	Women who had a painful burn/blistering skin reaction after 2 h of sun exposure as children/adolescents had a higher risk of vitiligo than those with no reaction or only redness after sun exposure. Women with strong tanning abilities had a higher risk of developing vitiligo compared to those without the ability to tan. In this study, no correlation was observed between the risk of vitiligo and body mass index, physical activity, or alcohol intake.	9
Kulkarni et al. (2016, India) (11)	A cross-sectional study to assess the incompatible dietary behaviors of patients suffering from skin diseases: A pilot study	32 patients with vitiligo aged 18–60 years with disease duration of up to 6 months, and 32 healthy controls	Incompatible diet	Case–control study	NR	The scores for consumption of incompatible diet and dietary habits in patients with vitiligo were similar to those of their respective controls.	7
Liu et al. (2021, China) (12)	Location, spreading, and oral corticosteroids are associated with insomnia in patients with vitiligo: a case–control study	Patients with vitiligo with insomnia, 204; patients with vitiligo without insomnia, 205	With or without insomnia	Case–control study	NR	Vitiligo on the face and neck, progression of vitiligo, and oral corticosteroids remained risk factors for insomnia in patients with vitiligo.	5
Lee et al. (2021, Korea) (13)	Height and risk of vitiligo: a nationwide cohort study	15,980,754 individuals	Height	Cohort study	Participants were followed until vitiligo diagnosis or until the end of 2015	Findings from this nationwide cohort study suggest that adult height positively correlates with the risk of vitiligo in Koreans. The association was stronger in the elderly population.	8
Sanad et al. (2020, Egypt) (14)	Serum zinc and inflammatory cytokines in vitiligo	Patients with vitiligo, 50; healthy controls, 100	Zinc	Case–control study	NR	The mean serum levels of zinc were significantly reduced in patients with vitiligo	7
Lee et al. (2020, Korea) (15)	Association between vitiligo and smoking: a nationwide population-based study in Korea	Patients with vitiligo, 22,811	Smoking	Cohort study	NR	The results suggested there are suppressive effects of smoking on the development of vitiligo.	8
Derakhshandeh-Rishehri et al. (2019, Iran) (16)	Role of fatty acid intake in generalized vitiligo	Patients with vitiligo, 100; controls, 110	Total fat, PUFA, MUFA, SFA, linoleic acid, linolenic acid, oleic acid, EPA, DHA, and cholesterol	Case–control study	NR	Total fat intake was associated with an increased risk of vitiligo.	7
Hussein et al. (2019, Egypt) (17)	Role of vitamin B12, folic acid, and oxidative damage in the serum of patients with vitiligo	Patients with vitiligo, 42; controls, 36	Vitamin B12 and folic acid	Case–control study	6 months	The results declared significant decreases in vitamin B12 and folic acid levels in patients with vitiligo compared to those in controls.	8
Iraji et al. (2017, Iran) (18)	Comparing the improvement of unstable vitiligo in patients treated by topical PUVA therapy alone, topical PUVA therapy and oral vitamin D, and topical PUVA therapy and oral vitamin D and vitamin B12	60 patients with active vitiligo	Vitamin D and B12	Randomized controlled trial	6 months	The group receiving vitamin D demonstrated higher reductions in the extent and area of lesions compared to that in the control group.	6
Akhter et al. (2017, Pakistan) (19)	Estimation of serum vitamin B12, folic acid, homocysteine, and ferritin levels in subjects with vitiligo	Patients with vitiligo, 50; controls, 50	Vitamin B12 and folic acid	Case–control study	12 months	Serum vitamin B12 and folic acid levels were significantly lower in patients with vitiligo than in controls.	8
Dass (2016, India) (20)	Search for clinical and laboratory markers of severity and instability of vitiligo: a cross-sectional observational hospital-based study	Patients with vitiligo, 40; controls, 40	Vitamin B12, folic acid, and Hcy	A cross-sectional observational study		Elevated serum Hcy levels and reduced serum vitamin B12 levels were significantly associated with vitiligo. No significant association was observed with reduced serum folic acid levels.	6
Wu et al. (2015, Providence) (21)	Use of permanent hair dyes and risk of vitiligo in women	254 incident vitiligo cases among 68,176 participants	Permanent hair dyes	Cohort study	NR	After adjusting for multiple covariates, there was a borderline increased risk of vitiligo associated with using permanent hair dyes. This association was more pronounced in individuals who used hair dyes for a longer duration, initiated use before the age of 30, and had a longer usage period since their first use.	8
Manisha et al. (2015, India) (22)	Epidemiological study of svitra (vitiligo) with special reference to viruddha ahara (incompatible diet)	Patients with vitiligo, 242	Incompatible diet	Observational studies	NR	The study observed that among the 242 patients with vitiligo, 100% had an incompatible combination of food, and 100% had an incompatible food sequence. In addition, 95.45% of patients exhibited an incompatible cooking method, and 71.90% reported an incompatibility of palatability. Based on the analysis of the data, the researchers concluded that incompatible food is the most potent etiological factor of vitiligo and should be avoided.	2
Ghiasi et al. (2015, Iran) (23)	Serum levels of vitamin B12, folic acid, and homocysteine in patients with vitiligo	Patients with vitiligo, 30; controls, 30	Vitamin B12 and folic acid	Case–control study	NR	No significant differences were observed in the levels of serum homocysteine, vitamin B12, and folic acid between patients with vitiligo and healthy controls. Moreover, there were no associations between these factors and age, body weight, or sex, nor with the extent, duration, and type of vitiligo.	7
Colucci et al. (2015, Italy) (24)	Evaluation of an oral supplement containing *Phyllanthus emblica* fruit extracts, vitamin E, and carotenoids in vitiligo treatment	Patients with vitiligo, 65; controls, 65	Group A included patients treated with oral antioxidants, and group B included individuals not treated with antioxidants. Group A patients took one tablet of an oral supplement containing *P. emblica* (100 mg), vitamin E (4.7 mg), and carotenoids (10 mg) three times a day for 6 months and were asked to stop the treatment in case of side effects. Both groups were treated at the same time with a comparable topical therapy and/or phototherapy	Case–control study	10 months	Group A patients showed significantly mild repigmentation in the head and neck region and on the trunk compared to other body sites. Although the repigmentation was not statistically significant for each individual body site, it was higher overall. Additionally, patients in Group A exhibited a higher level of disease stability.	6
Khurrum et al. (2014, Saudi Arabia) (25)	Is there a real relationship between serum levels of homocysteine and vitiligo?	Patients with vitiligo, 153; controls, 153	Vitamin B12, Hcy, and folic acid	Case–control study	NR	The results of this study revealed that there was no association between serum levels of Hcy and vitamin B12 and vitiligo. However, the folic acid levels were higher in patients with vitiligo than in controls.	6
Kim et al. (2015, Korea) (26)	Childhood facial vitiligo: How intractable is it?	Medical data and photos of 111 children with facial vitiligo who were followed up for more than 1 year.	Nutritional education, vitamin E (α-tocopherol 100–400 IU/day), folic acid (1–2 mg/day), multivitamin intake, and antioxidant cosmetics are the mainstay of treatment. Conventional therapies, including oral, topical, and/or intralesional corticosteroids, topical macrolactam, excimer lasers, and epidermal grafts, were employed	Single-arm experiment	NR	9% of patients demonstrated no improvement regardless of treatment modality, whereas 91% showed improvement in lesions.	2
Araujo et al. (2014, Brazil) (27)	The relation between vitamin B12 levels and vitiligo repigmentation	Thirty-three patients were treated for vitiligo lesions using either 308 nm excimer light or NB UVB 311 nm (depending on the expansion of the lesion). They were given vitamin B12 before treatment began.	Vitamin B12	Single-arm experiment	NR	An association between vitamin B12 levels (upper than 365) and better repigmentation was not found in any of the subjects.	1
Finamor et al. (2013, Brazil) (28)	A pilot study assessing the effect of prolonged administration of high daily doses of vitamin D on the clinical course of vitiligo and psoriasis	Serum 25(OH)D3, PTH	16 patients with vitiligo received vitamin D3 35,000 IU once daily for 6 months in association with a low-calcium diet (avoiding dairy products and calcium-enriched foods like oats, rice, or soy “milk”) and hydration (minimum 2.5 L daily)	Before–after study in the same patient	NR	Following the treatment, there was a significant increase in the levels of 25(OH)D3 and a significant decrease in the levels of PTH among patients with vitiligo. The serum concentrations of PTH and 25(OH)D3 were inversely correlated. Of the 16 patients with vitiligo, 14 achieved a repigmentation level ranging from 25% to 75%.	4
Yaghoobi et al. (2011, Iran) (29)	Original article title: “Comparison of therapeutic efficacy of topical corticosteroid and oral zinc sulfate-topical corticosteroid combination in the treatment of patients with vitiligo: a clinical trial”	35 patients with vitiligo were randomized into two groups, with the first group receiving topical corticosteroids and the second group receiving a combination of oral zinc sulfate-topical corticosteroids	Zinc	Randomized controlled trial	1 year	The mean response in the corticosteroid group was 21.43%, while in the zinc sulfate-corticosteroid combination group, it was 24.7%. However, there was no statistically significant difference between the two groups regarding therapeutic efficacy.	7
Silverberg et al. (2011, United States) (30)	Serum homocysteine is associated with the extent of vitiligo vulgaris	31 adult and 24 pediatric patients with vitiligo vulgaris	Homocysteine and vitamin B12	Cohort study	3 years	Active vitamin B12 supplementation may be beneficial for patients with vitiligo.	7
Gonul et al. (2010, Turkey) (31)	Serum vitamin B12, folate, ferritin, and iron levels in Turkish patients with vitiligo	Patients with vitiligo, 42; controls, 36	Vitamin B12 and folic acid	Case–control study	NR	The vitamin B12 and folate levels in patients with vitiligo did not differ from those of controls.	7
Khan et al. (2009, India) (32)	Circulatory levels of antioxidants and lipid peroxidation in Indian patients with generalized and localized vitiligo	Patients with vitiligo, 30; controls, 30	Vitamin C and vitamin E	Case–control study	NR	The vitamin C and E levels of the patients with vitiligo were significantly lower compared to those in the controls.	7
Mouzas et al. (2008, Greece) (33)	Increased frequency of self-reported parasomnias in patients suffering from vitiligo	Group A, 116 patients with vitiligo; Group B, 52 patients suffering from other dermatological disorders without psychogenic involvement (such as acne). The control group (Group C) consisted of 48 partners and relatives of the patients without dermatological disorders	Nocturnal enuresis, sleepwalking, night illusions, sleep terrors, and nightmares	Case–control study	NR	Vitiligo sufferers reported significantly more sleep disorders compared to that in the controls, especially sleepwalking, nocturnal enuresis, night illusions, sleep terrors, and nightmares. In contrast, individuals with other dermatological diseases showed a statistically significant difference compared to that in the control group only in nightmares and nocturnal enuresis. Additionally, when comparing vitiligo sufferers to those with other dermatological diseases, significant differences were observed in nightmares, night illusions, and sleepwalking. However, these two groups had no statistically significant difference in sleep terrors and nocturnal enuresis.	6
Agrawal et al. (2004, India) (34)	Study on the antioxidant status of patients with vitiligo at different age groups in Baroda	Patients with vitiligo, 63; controls, 60	Vitamin E	Case–control study	NR	No significant change in plasma vitamin E levels was observed in vitiliginous patients compared to that in controls.	6
Akyol et al. (2002, Turkey) (35)	The effects of vitamin E on the skin lipid peroxidation and the clinical improvement in patients with vitiligo treated with PUVA	Patients were assigned to receive either only PUVA (first group: 15) or PUVA and vitamin E (900 IU daily perorally) (second group: 15) for 6 months	Vitamin E	Case–control study	6 months	Vitamin E may prevent oxidative distress caused by PUVA therapy. However, it does not have a significant impact on the clinical improvement of vitiligo lesions.	5
TJIOE et al. (2002, Sweden) (36)	Erratum: Treatment of vitiligo vulgaris with narrow-band UVB (311 nm) for one year and the effect of the addition of folic acid and vitamin B12	Patients with vitiligo, 27	The first group received narrow-band UVB phototherapy, and the second group received vitamin B121000 mg sustained-release tablets and folic acid 5-mg tablets twice a day and received narrow-band UVB phototherapy.	Randomized controlled trial	12 months	The study reconfirmed the efficacy of narrow-band UVB phototherapy in vitiligo. However, it did not demonstrate any additional benefits from adding vitamin B12 and folic acid.	5
Picardo et al. (1994, Italy) (37)	Antioxidant status in the blood of patients with active vitiligo	Patients with vitiligo, 62; controls, 60	Vitamin E	Case–control study	NR	The blood levels of vitamin E in individuals with vitiligo were not significantly different from those of healthy age-matched controls.	6
Bashrahil et al. (2022, SAU) (38)	Association between vitamin D, zinc, and thyroid biomarker levels with vitiligo disease: a retrospective cohort study in a tertiary care center	Patients with vitiligo, 297	Vitamin D and zinc	Cohort study	NR	No significant association was observed between vitamin D or zinc levels and any of the characteristics or treatments of vitiligo.	5
Memon et al. (2021, Pakistan) (39)	Effect of vitamin B12 and folic acid in patients with vitiligo	Patients with vitiligo, 155	Vitamin B12 and folic acid	Cross-sectional study	6 months	Serum vitamin B12 and folic acid levels significantly affected the duration of vitiligo in patients.	5
Boisseau-Garsaud et al. (2002, France) (40)	Increase in total blood antioxidant status and selenium levels in black patients with active vitiligo	Patients with vitiligo, 11 Healthy controls, 11	Selenium	Case–control study	NR	Total blood antioxidant status and selenium levels were significantly increased in vitiligo patients, compared to those in sex- and age-matched controls	6

NR, not reported; NOS, Newcastle–Ottawa Scale; UVB, ultraviolet B-rays; Hcy, homocysteine; SFA, saturated fatty acid; MUFA, monounsaturated fatty acid; PUFA, polyunsaturated fatty acid; EPA, eicosapentaenoic acid; DHA, docosahexaenoic acid; PTH, parathormone.

NOS scores ranged from 0 to 9 points. A star scoring system was used to semi-quantitatively assess study quality. Each numbered item has been adjusted to a maximum of four stars in the selection and exposure categories. A maximum of two stars was assigned for comparison purposes. We considered studies achieving ≥7, 4–6, and <4 stars as having high, medium, and poor quality, respectively.

**Table 2:** Characteristics of the studies included in the meta-analysis and the Newcastle–Ottawa Scale (NOS) Quality Assessment Table.

Author (pub. year)	Study setting	Study period	Study design	Instruments used in the study	Controls: total number (M/F)	Cases: total number(M/F)	Classification of vitiligo	Mean age of controls, years, mean (SD)	Mean age of cases years, mean (SD)	Disease Duration, mean (SD)	NOS
Khoshdel et al. (2022) (41)	Iran	NR	Case–control study	zinc, copper	137 (62/75)	117 (48/69)	84% of patients had generalized/universal and 16% of localized/segmental stable vitiligo	47.19 (8.03)	37.64 (13.01)	9.57 (9.25)	8
Zaki et al. (2020) (42)	Egypt	1/2009–9/2009	Case–control study	zinc	50 (24/26)	50 (22/28)	Different types of vitiligo	31.19 (10.46)	33.64 (14)	NR	8
Wacewicz et al. (2018) (43)	Poland	NR	Case–control study	zinc, copper, selenium,	58 (17/41)	50 (21/29)	Active generalized vitiligo	40.12 (13.80)	44.76 (15.62)	NR	7
Mirnezami et al. (2018) (44)	Iran	4/2015–4/2016	Case–control study	zinc	103	103	Generalized, focal, and mucosal vitiligo	NR	NR	NR	7
Narang et al. (2017) (45)	India	NR	Case–control study	zinc, copper	8	29	NR	NR	NR	NR	6
Bagheri Hamidi et al. (2020) (46)	Iran	2/2017–4/2018	Case–control study	vitamin B12	100 (55/45)	104 (52/52)	Vulgaris, segmental, and universalis	35.97 (10.98)	35.63 (10.45)	11.66 (9.59)	7
Mogaddam et al. (2017) (47)	Iran	3/2012–4/2013	Case–control study	zinc	100 (54/46)	100 (54/46)	vulgaris	25.32 (2.47)	24.97 (2.58)	41.37 (26.41)	8
Dogan et al. (2016) (48)	Turkey	NR	Case–control study	zinc	52	52	NR	NR	NR	NR	5
Ataş et al. (2015) (49)	Turkey	2011–2013	Case–control study	folic acid, vitamin B12	60 (30/30)	60 (30/30)	Acrofacial, 8 Segmental, 3 Generalized, 48 Universal, 1	36.25 (7.8)	35.7 (11.2)	NR	7
Agrawal et al. (2014) (50)	Nepal	NR	Case–control study	vitamin C, vitamin E	80 (36/44)	80 (39/41)	Active and stable patients with vitiligo	32.61 (15.63)	32.66 (16.82)	8.76 (8.51)	7
Ramadan et al. (2013) (51)	Egypt	NR	Case–control study	vitamin E	15 (4/11)	15 (6/9)	the non-segmental type and only stable	33.47 (10.21)	30.53 (14.77)	6.27 (3.11)	7
Yasar et al. (2012) (52)	Turkey	NR	Case-control study	folic acid, vitamin B12	40 (22/18)	40 (23/17)	focal, segmental, acrofacial, and generalized	25.42 (4.48)	27.77 (13.44)	19.60 (13.39)	6
Karadag et al. (2012) (53)	Turkey	NR	Case–control study	vitamin B12, folic acid	52 (17/35)	69 (33/36)	NR	32.3 (16.7)	37.6 (8.6)	25.5 (35.3)	5
Balci et al. (2009) (54)	Turkey	NR	Case–control study	vitamin B12, folic acid	31 (14/17)	48 (27/21)	Vitiligo was clinically defined as localized, generalized, or universal, whereas disease activity was identified as stable or progressive	39.32 (13.15)	37.94 (16.27)	9.28 (9.32)	6
Jain et al. (2008) (55)	India	11/2006–11/2007	Case–control study	vitamin E	40 (20/20)	40 (20/20)	Generalized vitiligo	NR	NR	NR	6
Ines et al. (2006) (56)	Tunisia	NR	Case–control study	vitamin A, vitamin E, selenium	40 (25/15)	36 (22/14)	Active and stable patients with vitiligo	NR	NR	NR	7
Park et al. (2005) (57)	Korea	NR	Case–control study	vitamin B12, folic acid	80 (35/45)	77 (32/45)	Localized and generalized vitiligo	35.8 (11.8)	34.5 (18)	NR	7
Arora et al. (2002) (58)	India	9/1993–2/1995	Case−control study	zinc	24	15	NR	NR	NR	NR	6
Kim et al. (1999) (59)	Korea	NR	Case–control study	vitamin B12, folic acid	30 (14/16)	100 (50/50)	Eighty-seven patients had spreading vitiligo, and 13 were stable.	NR	NR	NR	6
Hussein et al. (2019) (17)	Egypt	1/11/2018–31/3/2019	Case–control study	vitamin B12, folic acid	36 (22/14)	42 (29/13)	Segmental, 30 non-segmental, 40	38.00 (4.81)	36.87 (11.09)	6.74 (0.80)	8
Sharma et al. (2017) (60)	India	NR	Case–control study	smoking, alcohol consumption	100 (64/36)	100 (66/34)	Non-segmental vitiligo	NR	NR	NR	8
Tanacan et al. (2020) (61)	Turkey	11/2014–3/2016	Cross-sectional study	smoking, alcohol consumption,	155 (84/71)	155 (83/72)	NR	37.37 (12.60)	37.04 (12.07)	NR	6
Dragoni et al. (2017) (62)	Italu	3/2012–3/2015	Case–control study	smoking	200 (92/108)	200 (92/108)	Non-segmental vitiligo	NR	NR	NR	6
Taneja et al. (2020) (63)	India	NR	Cross-sectional study	smoking	54 (22/33)	54 (19/35)	NR	32.4 (9.7)	30.7 (11.3)	10.3 (5.8)	7
Gorial et al. (2021) (64)	Iraq	9/2018–5/2019	Case–control study	smoking	63 (30/33)	63 (34/29)	Vulgaris, 45 Universal, 9 Focal, 7 Acrofacial, 2	39.9 (11.6)	38.7 (14.0)	13.3 (12.7)	7
Haider et al. (2010) (65)	Bangladesh	9/2007–7/2008	Case–control study	vitamin C, zinc	30 (12/18)	30 (10/20)	NR	NR	NR	NR	7
Barikbin et al. (2011) (66)	Iran	NR	Case–control study	selenium	45(15/30)	60 (26/34)	Active vitiligo vulgaris	31.28	31.83	NR	7
Ozturk et al. (2008) (67)	Turkey	NR	Case–control study	selenium	30 (12/18)	30 (19/11)	Generalized stable vitiligo	27.9 (7.1)	23.6 (7.4)	NR	7
Beazley et al. (1999) (68)	UK	NR	Case–control study	selenium	61 (19/42)	5932	Most of the group presented the clinical type of vitiligo vulgaris (n 44), and 13 patients had acrofacial vitiligo, two the segmental type, one the focal form, and one vitiligo totalis	NR	NR	NR	7
Yandong Wang et al. (2012) (69)	China	9/2010–9/2011	Case–control study	zinc, copper	120	120	NR	NR	NR	NR	7
Xuemin Wang (2011) (70)	China	NR	Case–control study	zinc, copper, iron	30	28	NR	NR	NR	NR	6
Aiping Yao (2011) (71)	China	1/2007–2009	Case–control study	zinc, copper, iron, selenium	50	90	Segmental vitiligo, 35 Localized vitiligo, 45 Generalized vitiligo, 10	36.7 (16.8)	35.6 (20.3)	NR	7
Jin Zhao et al. (2011) (72)	China	NR	Case–control study	selenium	16	36	Localized vitiligo, 6 Generalized vitiligo, 9 Disseminated vitiligo, 2 Segmental vitiligo, 1	28.45 (11.93)	36.4 (19.5)	NR	7
Zongping Li et al. (2001) (73)	China	NR	Case–control study	copper	30	96	NR	NR	NR	NR	6
Fei Wang et al. (1993) (74)	China	NR	Case–control study	zinc, copper, iron, selenium	34	34	NR	NR	NR	NR	8
Yi Wu et al. (2010) (75)	China	NR	Case–control study	zinc, copper	70	70	NR	NR	NR	NR	7
Xiaohua Wang et al. (1996) (76)	China	NR	Case–control study	zinc, copper	141	48	NR	NR	27.8 (9.7)	NR	7
Caixia Tu et al. (1991) (77)	China	NR	Case–control study	zinc, copper	36	27	Localized vitiligo generalized vitiligo	NR	NR	NR	7
Caixia Tu et al. (1998) (78)	China	NR	Case–control study	selenium	37	29	Segmental vitiligo, 5 Localized vitiligo, 13 Generalized vitiligo, 11	NR	NR	NR	7
Jialing Song et al. (2017) (79)	China	6/2013–1/2016	Case–control study	zinc, copper, selenium	63	63	NR	37.02 (5.91)	37.22 (5.63)	NR	7
Al-Hattab et al. (2020) (80)	Iraq	11/2019–3/2020	Case–control study	zinc, vitamin c	50 (24/26)	50 (28/22)	NR	26.30 (8.11)	28.60 (8.00)	NR	7

NOS, Newcastle–Ottawa Scale; NR, not reported; Pub, public. A star scoring system was used to semi-quantitatively assess study quality. Each numbered item was adjusted to a maximum of four stars in the selection and exposure categories. A maximum of two stars was assigned for comparison purposes. NOS scores ranged from 0 to 9 points. We considered studies achieving ≥7 stars as high quality, those with 4–6 stars as medium quality, and those with <4 stars as poor quality.

### Smoking

A meta-analysis of five studies (60–64) involving 552 patients indicated that the number of smokers in the vitiligo group was higher than in the control group [mean difference (MD), 1.240; 95% CI, 1.057–1.455; p <0.05; **Table 3**; **Supplementary Figure S5**). Additionally, a cohort study (15) involving 22,991,641 patients showed that smoking has an inhibitory effect on the development of vitiligo.

**Table 3:** The number of people with smoking and alcohol consumption habits between the vitiligo and control groups.

Studies	Vitiligo group		Control group		RR [95% CI]	p-value
	Events	Total	Events	Total		
Smoking						
Sharma et al. (2017) (60)	25	100	19	100	1.316 [0.776–2.231]	
Tanacan et al. (2020) (61)	59	155	56	155	1.054 [0.788–1.408]	
Dragoni et al. (2017) (62)	72	180	58	200	1.379 [1.041–1.827]	
Taneja et al. (2020) (63)	8	54	9	54	0.889 [0.371–2.131]	
Gorial et al. (2021) (64)	43	63	31	63	1.387 [1.025–1.876]	
Meta-analysis (fixed, I^2^ = 0%)					1.240 [1.057–1.455]	0.008^#^
Alcohol consumption						
Sharma et al. (2017) (60)	34	100	27	100	1.259 [0.825–1.921]	
Tanacan et al. (2020) (61)	50	155	30	155	1.667 [1.124–2.472]	
Meta-analysis (random, I^2^ = 0%)					1.474 [1.105–1.965]	0.008^#^

CI, confidence interval; RR, risk ratio; ^#^p <0.005.

### Alcohol consumption

We conducted a meta-analysis of two studies (60, 61) involving 255 patients, and the results showed that there was a significantly higher prevalence of alcohol dependence among vitiligo patients compared to the control group (MD, 1.474; 95% CI, 1.105–1.965; p <0.005; **Table 4**; **Supplementary Figure S6**).

**Table 4:** Serum zinc, vitamin B12, folic acid, vitamin E, and vitamin C levels between the vitiligo and control groups.

Studies	Vitiligo group		Control group		MD [95% CI]	p-value
	Mean	SD	Mean	SD		
1. Zinc						
Khoshdel et al. (2022) (41)	95.01	58.95	121.83	33.8	−0.570 [−0.821–−0.318]	
Zaki et al. (2020) (42)	50.93	11.02	77.09	12.16	−2.254 [−2.758–−1.751]	
Wacewicz et al. (2018) (43)	0.848	0.12	0.997	0.292	−0.650 [−1.038–−0.262]	
Mirnezami et al. (2018) (44)	85.4	14.1	91.8	16.2	−0.421 [−0.698–−0.144]	
Narang et al. (2017) (45)	20.05	7.89	32.72	0.68	−1.794 [−2.682–−0.905]	
Mogaddam et al. (2017) (47)	80.11	17.1	96.1	16.16	−0.961 [−1.254–−0.668]	
Dogan et al. (2016) (48)	92.84	15.51	88.94	13.43	0.269 [−0.117–0.655]	
Arora et al. (2002) (58)	97.3	26.6	105.3	30.1	−0.278 [−0.926–0.371]	
Haider et al. (2010) (65)	1.08	0.07	0.95	0.35	0.515 [0.000–1.030]	
Jialing Song et al. (2017) (79)	0.84	0.12	1.1	0.2	−1.576 [-1.980–−1.173]	
Caixia Tu et al. (1991) (77)	11.5	3.15	13.54	2.34	−0.751 [−1.268–−0.235]	
Xiaohua Wang et al. (1996) (76)	74.23	18.99	97.4	13.8	−1.517 [−1.879–−1.155]	
Yi Wu et al. (2010) (75)	6.416	1.758	7.193	1.412	−0.487 [−0.824–−0.151]	
Fei Wang et al. (1993) (74)	0.9	0.51	1.06	2.25	−0.098 [−0.574–0.378]	
Aiping Yao et al. (2011) (71)	0.88	0.26	1.07	0.31	−0.682 [−1.036–−0.327]	
Yandong Wang et al. (2012) (69)	12.79	2.31	13.02	3.53	−0.077 [−0.330–0.176]	
Xuemin Wang et al. (2011) (70)	9.9	0.51	15.62	2.94	−2.667 [−3.380–−1.953]	
Al-Hattab et al. (2020) (80)	82.49	23.92	98.78	36.62	−0.527 [−0.926–−0.128]	
Meta-analysis (random, I^2^ = 91.3%)					−0.774 [−1.083–−0.466]	0.000^*^
1.1 Vitiligo type						
1.1 Generalized vitiligo						
Mirnezami et al. (2018) (44)	81.3	12.7	91.8	16.2	−0.701 [−1.024–−0.379]	
Khoshdel et al. (2022) (41)	93.11	59.33	121.83	33.8	−0.624 [−0.891–−0.357]	
Yi Wu et al. (2010) (75)	5.401	1.198	7.193	1.412	−1.306 [−1.859–−0.752]	
Meta-analysis (random, I^2^ = 58.3%)					−0.799 [−1.121–−0.477]	0.000^*^
1.1.2 Focal vitiligo						
Mirnezami et al. (2018) (44)	92.1	13.8	91.8	16.2	0.019 [−0.349–0.388]	
Khoshdel et al. (2022) (41)	98.69	58.63	121.83	33.8	−0.610 [−1.075–−0.146]	
Yi Wu et al. (2010) (75)	6.767	1.793	7.193	1.412	−0.269 [−0.629–0.092]	
Meta-analysis (random, I^2^ = 54.3%)					−0.264 [−0.601–0.073]	0.125
1.2 Sex						
1.2.1 Female						
Khoshdel et al. (2022) (41)	92.55	56.83	112.53	33.78	−0.438 [−0.769–−0.107]	
Wacewicz et al. (2018) (43)	0.812	0.101	0.987	0.281	−0.778 [−1.271–−0.284]	
Meta-analysis (fixed, I^2^ = 20.3%)					−0.557 [−0.874–−0.240]	0.001^*^
1.2.2 Male						
Khoshdel et al. (2022) (41)	99.12	62.1	126.87	29.28	−0.597 [−0.982–−0.212]	
Wacewicz et al. (2018) (43)	0.897	0.129	1.021	0.325	−0.523 [−1.174–0.128]	
Meta-analysis (fixed, I^2^ = 0.0%)					−0.578 [−0.909–−0.246]	0.0000^*^
1.3 Vitiligo disease activity						
1.3.1 Progressive						
Jialing Song et al. (2017) (79)	0.67	0.1	1.1	0.2	−2.556 [−3.089–−2.024]	
Yandong Wang et al. (2012) (69)	13.2	3.44	13.02	3.53	0.051 [−0.266–0.369]	
Meta-analysis (random, I^2^ = 98.5%)					−1.243 [−3.798–1.312]	0.340
1.3.2 Stable						
Jialing Song et al. (2017) (79)	0.95	0.14	1.1	0.2	−0.804 [−1.306–−0.303]	
Yandong Wang et al. (2012) (69)	12.83	2.86	13.02	3.53	−0.057 [−0.361–0.246]	
Meta-analysis (random, I^2^ = 84.8%)					−0.403 [−1.133–0.327]	0.279
2.Vitamin B12						
Bagheri Hamidi et al. (2020) (46)	384.62	198.63	434.1	177.86	−0.262 [−0.538–0.014]	
Ataş et al. (2015) (49)	372	142	348	121	0.182 [−0.177–0.541]	
Yasar et al. (2012) (52)	212.9	81.67	241.15	126.23	−0.266 [−0.706–0.175]	
Karadag et al. (2012) (53)	250.6	112.4	316.5	152	−0.504 [−0.869–−0.138]	
Balci et al. (2009) (54)	211.69	211.38	198.32	103.49	0.075 [−0.376–0.527]	
Park et al. (2005) (57)	668	290	875	302	−0.699 [−1.021–−0.376]	
Kim et al. (1999) (59)	630.25	230.94	627.16	251.35	0.013 [−0.395–0.421]	
Hussein et al. (2019) (17)	186	11.5	399.23	19	−13.751 [−16.012–−11.491]	
Meta-analysis (random, I^2^ = 94.8%)					−0.951 [−1.672–−0.275]	0.006^#^
3. Folic acid						
Ataş et al. (2015) (49)	9.8	2.9	10.2	2.7	−0.400 [−1.403–0.603]	
Yasar et al. (2012) (52)	6.59	2.78	5.39	2.41	1.200 [0.060–2.340]	
Karadag et al. (2012) (53)	7.5	3.1	7	2.2	0.500 [−0.445–1.445]	
Balci et al. (2009) (54)	6.14	2.45	6.25	3.44	−0.110 [−1.505–1.285]	
Kim et al. (1999) (59)	6.31	2.82	6.11	3.11	0.200 [−1.043–1.443]	
Hussein et al. (2019) (17)	1.22	0.2	11.33	0.67	−10.110 [−10.337–−9.883]	
Meta-analysis (random, I^2^ = 99.6%)					−1.463 [−7.133–4.208]	0.613
4.Vitamin E						
Ramadan et al. (2013) (51)	1.04	0.22	5.21	0.5	−10.796 [−13.712–−7.879]	
Jain et al. (2008) (55)	0.7	0.43	1.13	0.57	−0.852 [−1.310–−0.394]	
Ines et al. (2006) (56)	9.43	7.98	9.18	9.87	0.028 [−0.423–0.478]	
Agrawal et al. (2014) (50)	0.67	0.22	0.66	0.15	0.053 [−0.257–0.363]	
Meta-analysis (random, I^2^ = 93.8%)					−1.408 [−2.611–−0.206]	0.022^#^
5.Vitamin C						
Haider et al. (2010) (65)	25.01	7.14	25.94	7.98	−0.123 [−0.629–0.384]	
Agrawal et al. (2014) (50)	0.65	0.15	0.63	0.14	0.138 [−0.172–0.448]	
Al-Hattab et al. (2020) (80)	6.27	2.65	10.83	5.52	−1.053 [−1.472–−0.634]	
Meta-analysis (random, I^2^ = 90.2%)					−0.342 [−1.090–0.407]	0.371
6.Copper						
Khoshdel et al. (2022) (41)	113.57	59.43	138.9	38.14	−0.516 [−0.767–−0.265]	
Narang et al. (2017) (45)	31.7	10.28	22.52	1.95	0.994 [0.177–1.811]	
Wacewicz et al. (2018) (43)	1.099	0.273	1.038	0.336	0.198 [−0.181–0.577]	
Jialing Song et al. (2017) (79)	0.8	0.12	1.15	0.23	−1.908 [−2.334–−1.482]	
Caixia Tu et al. (1991) (77)	13.05	2.74	14.08	2.33	−0.410 [−0.914–0.094]	
Xiaohua Wang et al. (1996) (76)	107.6	10.16	109.2	16.7	−0.104 [−0.432–0.223]	
Yi Wu et al. (2010) (75)	1.46	0.471	1.536	0.345	−0.184 [−0.516–0.148]	
Fei Wang et al. (1993) (74)	0.88	0.17	1.13	0.21	−1.309 [−1.834–−0.783]	
Aiping Yao et al. (2011) (71)	0.69	0.15	1.12	0.2	−2.538 [−2.995–−2.080]	
Yandong Wang et al. (2012) (69)	13.1	2.56	14.78	2.4	−0.677 [−0.937–−0.417]	
Xuemin Wang et al. (2011) (70)	18.95	0.39	19.35	4.32	−0.128 [−0.644–0.387]	
Zongping Li et al. (2001) (73)	0.807	0.143	1.091	0.181	−1.859 [−2.330–−1.389]	
Meta-analysis (random, I^2^ = 94.2%)					−0.719 [−1.185–−0.252]	0.003^#^
6.1 Vitiligo disease activity						
6.1.1 Progressive						
Jialing Song et al. (2017) (79)	0.65	0.1	1.1	0.2	−2.675 [−3.219–−2.131]	
Yandong Wang et al. (2012) (69)	13.18	2.68	13.02	3.53	0.049 [−0.269–0.366]	
Meta-analysis (random, I^2^ = 98.6%)					−1.304 [−3.973–1.365]	0.338
6.1.2 Stable						
Jialing Song et al. (2017) (69)	1.04	0.17	1.1	0.2	−0.311 [−0.800–0.177]	
Yandong Wang et al. (2012) (79)	12.9	3.28	13.02	3.53	−0.035 [−0.338–0.269]	
Meta-analysis (fixed, I^2^ = 0.0%)					−0.112 [−0.369–0.146]	0.396
6.2 Sex						
6.2.1 Female						
Khoshdel et al. (2022) (41)	112.95	56.32	146.22	34.76	−0.718 [−1.055–−0.380]	
Wacewicz et al. (2018) (43)	1.128	0.317	1.118	0.348	0.030 [−0.446–0.505]	
Meta-analysis (random, I^2^ = 84.2%)					−0.364 [−1.095–0.368]	0.330
6.2.2 male						
Khoshdel et al. (2022) (41)	114.65	63.88	135.46	25.13	−0.451 [−0.832–−0.069]	
Wacewicz et al. (2018) (43)	1.058	0.196	0.845	0.21	1.053 [0.369–1.737]	
Meta-analysis (random, I^2^ = 92.9%)					0.273 [−1.199–1.745]	0.716
7. Selenium						
Wacewicz et al. (2018) (43)	51.3	13.99	79.42	18.97	−1.669 [−2.109–−1.229]	
Barikbin et al. (2011) (66)	1.021	0.04	0.909	0.01	3.616 [2.989–4.243]	
Ozturk et al. (2008) (67)	122.333	30.173	120.766	21.802	0.060 [−0.447–0.566]	
Beazley et al. (1999) (68)	1.27	0.32	0.93	0.2	1.687 [1.433–1.941]	
Caixia Tu et al. (1998) (78)	99.41	14.93	105.24	14.92	−0.391 [−0.881–0.100]	
Jialing Song et al. (2017) (79)	0.11	0.02	0.16	0.05	−1.313 [−1.702–−0.924]	
Fei Wang et al. (1993) (74)	0.1	0.02	0.13	0.14	−0.300 [−0.778–0.178]	
Jin Zhao et al. (2011) (72)	121.9	46.16	129.27	23.67	−0.181 [−0.771–0.409]	
Aiping Yao et al. (2011) (71)	0.09	0.03	0.14	0.07	−1.038 [−1.405–−0.671]	
Ines et al. (2006) (56)	1.37	0.19	0.93	0.07	3.138 [2.461–3.815]	
Meta-analysis (random, I^2^ = 98.2%)					0.350 [−0.687–1.387]	0.508
7.1 Vitiligo disease activity						
7.1.1 Progressive						
Jialing Song et al. (2017) (79)	0.06	0.01	0.16	0.05	−2.529 [−3.059–−1.999]	
Jin Zhao et al. (2011) (72)	121.08	44.83	129.27	23.67	−0.225 [−0.900–0.451]	
Meta-analysis (random, I^2^ = 98.0%)					−1.387 [−3.644–0.871]	0.229
7.1.2 Stable						
Jialing Song et al. (2017) (79)	0.12	0.03	0.16	0.05	−0.875 [−1.379–−0.370]	
Jin Zhao et al. (2011) (72)	122.72	48.74	129.27	23.67	−0.168 [−0.842–0.507]	
Meta-analysis (random, I^2^ = 63.1%)					−0.558 [−1.247–0.131]	0.112
8. Iron						
Fei Wang et al. (1993) (74)	2.37	0.78	1.35	0.51	1.548 [1.004–2.092]	
Aiping Yao et al. (2011) (71)	2.28	0.61	1.37	0.46	1.621 [1.226–2.016]	
Xuemin Wang et al. (2011) (70)	25.67	7.92	23.09	6.8	0.350 [−0.169–0.870]	
Meta-analysis (random, I^2^ = 89.8%)					1.181 [0.390–1.972]	0.003^#^

CI, confidence interval; RR, risk ratio; ^#^p < 0.005; *p <0.001.

### Diet

#### Vitamin C

A meta-analysis of three studies (50, 65, 80) showed no significant differences in serum vitamin C levels between patients with vitiligo and controls (MD, 0.342; 95% CI, −1.090–0.407; p >0.05; **Table 4**; **Supplementary Figure S7**). Another study (32) concluded that patients with vitiligo had significantly lower vitamin C levels than the controls.

#### Vitamin B12 and folic acid

One study (9) suggested combining folic acid and vitamin B12 supplementation with sunlight-induced repigmentation to be more effective than vitamin or sunlight exposure alone. In addition, two case–control studies (17, 19) reported that serum folic acid and vitamin B12 levels were significantly lower in patients with vitiligo than in controls.

A cross-sectional study (20) and a cohort study (30) showed that elevated serum homocysteine (Hcy) and reduced serum vitamin B12 levels were significantly associated with vitiligo. However, another two studies (23, 31) reported no significant differences in vitamin B12 and folic acid levels between patients with vitiligo and controls. In addition, one study (25) showed that patients with vitiligo had no significant difference in Hcy and vitamin B12 levels compared to that in controls. In contrast, those with vitiligo had higher folic acid levels.

An evaluation of 33 patients treated for vitiligo did not reveal an association between vitamin B12 levels and improved repigmentation (27). Tjioe et al. (36) reported that adding vitamin B12 and folic acid did not provide any therapeutic benefit in treating patients with vitiligo. Memon et al. (39) concluded that the serum levels of vitamin B12 and folic acid significantly affected the course of vitiligo.

A meta-analysis of eight studies (17, 46, 49, 52–54, 57, 59) showed that vitamin B12 levels were significantly lower in patients with vitiligo than in controls (MD, −0.951; 95% CI, −1.672–−0.275; p <0.05; **Table 4**; **Supplementary Figure S8**). In contrast, a meta-analysis of six studies (17, 49, 52–54, 59) showed no significant difference in the folate levels between patients with vitiligo and controls (MD, −1.463; 95% CI, −7.133–4.208; p >0.05; **Table 4**; **Supplementary Figure S9**).

#### Vitamin D

A randomized controlled trial (18) showed that vitamin D treatment resulted in a more significant reduction in the extent and size of lesions in patients with vitiligo than in controls, suggesting that vitamin D plays a role in preventing the progression of active vitiligo. Another study (28) concluded that high-dose vitamin D3 therapy (35, 000 IU once daily) is safe and effective for patients with vitiligo. A retrospective cohort study (38) reported no significant association between vitamin D and any feature or treatment of vitiligo. Among the studies, two research papers (18, 28) that found vitamin D treatment effective for vitiligo included a total of 76 patients. Meanwhile, one cohort study (38) that deemed it ineffective included 297 patients.

#### Vitamin E

Patients with vitiligo who received oral antioxidants (*Phyllanthus emblica*, vitamin E, and carotenoids) had significantly milder repigmentation of the head, neck, and trunk, along with a higher level of disease stability, compared to the corresponding patients who did not receive oral antioxidants (24).

A study (26) evaluated the long-term treatment of children with facial vitiligo. The study employed a combination of approaches, including nutrition education, vitamin E, folic acid, multivitamin intake, and antioxidant cosmetics, as the primary treatment. Additionally, conventional therapies were used as part of the treatment protocol. A total of 91% of patients demonstrated lesion improvement.

A case–control study (32) concluded that the serum vitamin E levels were significantly lower in patients with vitiligo than in controls. In contrast, two other case–control studies (34, 37) reported no significant difference in blood vitamin E levels in patients with vitiligo compared to age-matched healthy controls. Akyol et al. (35) concluded that vitamin E prevented oxidative distress caused by Psoralen UVA rays (PUVA) treatment; however, it did not affect the clinical improvement of vitiligo lesions. A meta-analysis of four studies (50, 51, 55, 56) showed that the serum VE levels were significantly lower in patients with vitiligo than in controls (MD, −1.408; 95% CI, −2.611–−0.206; p <0.05; **Table 4**; **Supplementary Figure S10**).

#### Zinc

A retrospective cohort study reported no significant association between zinc and any feature or treatment associated with vitiligo (38). A randomized controlled trial showed that although the group that received oral zinc sulfate combined with topical corticosteroids responded better than the group that received topical corticosteroids alone, there was no statistical difference (29). Another study suggested that the average zinc level in the serum of patients with vitiligo was significantly lower (14). The meta-analysis of 18 studies (41–45, 47, 48, 58, 65, 69–71, 74–77, 79, 80) showed that the serum zinc levels were significantly lower in patients with vitiligo than in controls (MD, −0.774; 95% CI, −1.083–−0.466; p <0.001; **Table 4**; **Supplementary Figure S11**). Three studies showed that the serum zinc levels were significantly lower in patients with generalized vitiligo than in healthy controls (MD, −0.799; 95% CI, −1.121–−0.477; p <0.001; **Table 4**; **Supplementary Figure S12**), while the serum zinc levels in patients with localized vitiligo were not different from those in the control group (MD, −0.264; 95% CI, −0.601–−0.073; p >0.05; **Table 4**; **Supplementary Figure S13**). Both female (MD, −0.557; 95% CI, −0.874–−0.240; p <0.005; **Table 4**; **Supplementary Figure S14**) and male (MD, −0.578; 95% CI, −0.909–−0.246; p <0.001; **Table 4**; **Supplementary Figure S15**) patients with vitiligo had significantly lower serum zinc levels than those of healthy controls. However, the opposite was true for the serum zinc levels in patients with progressive (MD, −1.243; 95% CI, −3.789–1.312; p >0.05; **Table 4**; **Supplementary Figure S16**) and stable (MD, −0.403; 95% CI, −1.133–0.327; p >0.05; **Table 4**; **Supplementary Figure S17**) vitiligo.

#### Incompatible diet

Incompatible diets refer to incorrect combinations of food components in formulations, insufficient or excessive processing, inappropriate consumption amounts, and/or eating at incorrect times of the day and/or in the wrong seasons (11). A case–control study (11) concluded that the mean composite scores of two questionnaires for assessing incompatible dietary habits in patients with vitiligo were similar to those of controls. Additionally, a study (22) revealed that patients with vitiligo must avoid incompatible foods, as these are the most potent causative factors for vitiligo.

#### Total fat intake

A previous study (16) highlighted that the quantity of total fat consumed in the diet had a greater impact on the risk of vitiligo compared to specific subclasses of fat. The study suggested that a high-fat diet increases the risk of developing vitiligo.

#### Copper

The meta-analysis of 12 studies (41, 43, 45, 69–71, 73–77, 79) showed that the serum copper levels were significantly lower in patients with vitiligo than in controls (MD, −0.719; 95% CI, −1.185–−0.252; p <0.005; **Table 4**; **Supplementary Figure S18**). Whether progressive (MD, −1.304; 95% CI, −3.973–1.365; p >0.05; **Table 4**; **Supplementary Figure S19**) or stable (MD, −0.112; 95% CI, −0.369–0.146; p >0.05; **Table 4**; **Supplementary Figure S20**), and male (MD, 0.273; 95% CI, −1.199–1.745; p >0.05; **Table 4**; **Supplementary Figure S21**) or female (MD, −0.364; 95% CI, −1.095–0.368; p >0.05; **Table 4**; **Supplementary Figure S22**) patients, there were no significant differences in the serum copper levels in patients with vitiligo compared to those in controls.

#### Selenium

The meta-analysis of 10 studies (43, 56, 66–68, 71, 72, 74, 78, 79) indicated no significant difference in the serum selenium levels between patients with vitiligo and controls (MD, 0.350; 95% CI, −0.687–1.387; p >0.05; **Table 4**; **Supplementary Figure S23**). No significant difference was observed in the serum selenium level between the control group and the patients with progressive (MD, −1.387; 95% CI, −3.644–0.871; p >0.05; **Table 4**; **Supplementary Figure S24**) and stable (MD, −0.558; 95% CI, −1.247–0.131; p >0.05; **Table 4**, **Supplementary Figure S25**) vitiligo.

#### Iron

The meta-analysis of three studies (70, 71, 74) showed that serum iron levels were significantly higher in patients with vitiligo than in controls (MD, 1.209; 95% CI, 0.403–2.014; p <0.0055; **Table 4**; **Supplementary Figure S26**).

### Exercise

No studies have examined the relationship between physical exercise and disease progression in patients with vitiligo.

### Tanning ability

Women who had a painful burn/blistering skin reaction after 2 h of sun exposure as children/adolescents had a higher risk of vitiligo than those with no reaction or only redness after sun exposure. Women with strong tanning abilities had a higher risk of developing vitiligo compared to those without the ability to tan (10).

### Sleeping

Two studies (12, 33) have examined the relationship between vitiligo and sleep disorders. One study indicated that patients with vitiligo reported significantly more sleep disturbances compared to that in controls, especially sleepwalking, nocturnal enuresis, nocturnal hallucinations, sleep fears, and nightmares. In addition, patients with vitiligo had statistically significant levels of nightmares, nocturnal hallucinations, and sleepwalking compared to those with other skin diseases; however, they did not have statistically significant levels of sleep phobias or nocturnal enuresis. Another study indicated that facial and neck vitiligo, vitiligo progression, and oral glucocorticoid use were risk factors for insomnia in patients with vitiligo.

### Permanent hair dyes

The previous use of permanent hair dyes increased the risk of vitiligo. The association with vitiligo was more pronounced in those who had used hair dyes for a longer duration, initiated their use before the age of 30 years, and had a longer period of usage since their first use (21).

### Height

A nationwide cohort study showed that height is positively associated with the risk of vitiligo in Korean adults. This association was stronger in the older adult population (age ≥65 years) (13).

### Sexual dysfunction

In our previous study (96), we observed a higher risk of sexual dysfunction in patients with vitiligo, with the relationship being more prominent in women than in men.

## Discussion

The importance of reviewing lifestyle habits (including smoking, alcohol consumption, diet, exercise, and sleep) lies in the provision of adjunctive measures for the treatment of vitiligo. Current treatments for vitiligo are abundant, with traditional approaches focusing on the autoimmune hypothesis through immunomodulatory and anti-inflammatory approaches. However, in recent years, new therapies, such as molecular targeted therapy, have become available (5–7). Despite the availability of various treatments, a few patients continue to experience unsatisfactory results with conventional therapies, and the effectiveness of newer treatments remains uncertain. This study carries several significant implications in this context.

We reviewed the smoking and alcohol consumption data of patients with vitiligo and observed that more patients with vitiligo smoked and drank alcohol compared to that in controls, which is contrary to the results of another cohort study (15), which suggested that smoking had a suppressive effect on the development of vitiligo. To date, there are no studies demonstrating the effect of smoking and alcohol consumption on the development of vitiligo.

Regarding diet, we focused on vitamins C, D, E, and B12, folic acid, zinc, copper, iron, selenium, incompatible diets, and total fatty acids. We observed no significant difference in the serum vitamin C levels between patients with vitiligo and controls, which refutes statements recommending vitamin C supplementation in patients with vitiligo. In addition, although the oxidative stress theory has been mentioned in studies on the pathogenesis of vitiligo, it is worth noting that vitamin C, as an antioxidant, may exert inhibitory effects on tyrosinase activity by inducing cytoplasmic acidification (95). This mechanism may contribute to a reduction in melanin content, offering a potential avenue for therapeutic intervention in vitiligo (58).

The results of this meta-analysis showed that vitamin B12 levels were low in patients with vitiligo. In contrast, the folic acid levels did not significantly differ from those of the controls. The folic acid and vitamin B12 levels are associated with homocysteine synthesis, inhibiting tyrosinase and reducing melanin production (81). Therefore, we recommend that vitamin B12 supplementation inhibits tyrosinase and reduces associated effects.

Decreased vitamin D levels play a role in the development of vitiligo by affecting Th1- and Th17-related immune responses (82). In contrast, studies have reported no role of circulating vitamin D in the pathogenesis of vitiligo. Although studies have suggested that vitamin D has a therapeutic effect on vitiligo, not all of these were single-drug studies, a few of these combined vitamin D with other therapeutic methods; therefore, the evidence for this is insufficient.

Our results suggested that vitamin E has a therapeutic effect on vitiligo; however, further studies are needed to confirm this hypothesis. Vitamin E, an antioxidant, inhibits tyrosinase activity, and its derivatives inhibit melanogenesis in epidermal melanocytes *in vitro*. In addition, vitamin E increases the expression of endosomal docking/fusion proteins, and melanosomes can be degraded within the lysosomal compartment by docking with lysosomes, decreasing the number of melanosomes (83, 84). Although the results of our meta-analysis identified low serum vitamin E levels in patients with vitiligo, these were inconclusive owing to the limited sample sizes and the observational nature of the included studies. Thus, whether vitamin E supplementation is beneficial for vitiligo needs to be confirmed in additional studies with larger sample sizes. Vitamin E supplementation is only appropriate for patients with vitamin E deficiency.

Copper, one of the trace elements, is a cofactor involved in the synthesis of melanin by tyrosinase and in the biosynthesis of superoxide dismutase, which plays an important role in protecting cells from oxidative stress (85, 86). Our study showed that patients with vitiligo had significantly lower serum copper levels than those of healthy controls. This indicates that copper may be involved in the pathogenesis of vitiligo, and further studies are needed to explain this.

Selenium is an essential immune nutrient for the human body, and the organic forms of selenium naturally exist in the human body: selenocysteine and selenoprotein. Glutathione peroxidase is the main selenium protein in the body, which helps control the excess production of free radicals at inflammatory sites (87). Our study did not find a difference in serum selenium between patients with vitiligo and healthy individuals. In the future, we can study the lesion site of vitiligo and observe whether the results have changed.

Toxic damage to melanocytes by redox-generated free radicals is one of the doctrines of the pathogenesis of vitiligo. Iron, an essential element for many important cellular functions in all organisms, catalyzes the formation of potentially toxic free radicals (88). Our research supports this view, although the results of two studies (31, 89) are diametrically opposed to ours.

Zinc is an antioxidant that inhibits apoptosis and may inhibit the apoptosis of melanocytes. Moreover, zinc plays an important role in the final stage of melanin formation. Therefore, zinc may have an important effect on vitiligo (43). The results of this meta-analysis showed low serum zinc levels in patients with vitiligo.

The association between incompatible diets and vitiligo remains controversial and poorly understood. High-fat diets are thought to increase the risk of vitiligo, and diets high in saturated fats have deleterious effects on macrophage phagocytosis and natural killer cell activity in autoimmune diseases. Moreover, it has been well established that high-fat diets can contribute to the development of various diseases and have detrimental effects on the lifespan of animals (16).

The benefits of gluten-free diets for vitiligo have been reported in only two cases. Although chronic physical exercise can change the balance of inflammation to an anti-inflammatory state and improve the structure and function of the endogenous antioxidant system and mitochondria (90), thus improving the clinical symptoms and quality of life of patients with vitiligo, observational and experimental studies are still lacking. Moreover, vitiligo has been consistently linked to sleep disorders, and extensive research indicates the presence of a cyclic relationship between vitiligo and depression, in which sleep deprivation may play a contributing role (12).

Women with a strong ability to tan reportedly present at a higher risk of developing vitiligo. This association may be attributed to various factors, including the direct impact of UV rays on DNA, leading to the upregulation of the tyrosinase gene, and the direct influence of UV rays on melanin cell membranes. These mechanisms contribute to the overall tanning response observed in individuals (91). Therefore, tyrosinase seems to be a key player in the tanning response (92). A recent study (93) published in *The Lancet* indicated that regions with a higher overall prevalence of vitiligo are located in South Asia, including India, Bangladesh, Nepal, Pakistan, and Bhutan. This may be related to the greater visibility of vitiligo in individuals with darker skin tones.

Permanent hair dye contains many chemicals, including phenols such as p-aminophenol and resorcinol (94). Phenols act as tyrosinase analogs and interfere with melanin production, which may be associated with an increased risk of vitiligo caused by permanent hair dye use (21).

A national cohort study in South Korea identified a significant association between height and an increased risk of vitiligo in Korean adults (13). However, data from other countries in Asia, Europe, and the United States is lacking. In the future, large cohort and mechanistic studies on the relationship between height and vitiligo should be conducted to further explore this phenomenon.

### Limitations

This study had certain limitations. First, the available literature on the lifestyle habits of patients with vitiligo is relatively limited, resulting in insufficient evidence in certain areas. Second, the studies in this review were highly heterogeneous. Third, most of the studies were conducted in Asian and African countries, and there is a lack of data from studies conducted in Europe and the United States. Fourth, there were limited data available for the meta-analysis and bias assessment. Fifth, most of the data came from non-segmental vitiligo cases, and the evidence for segmental vitiligo was weak. Moreover, the limited data in this study could not conduct a more in-depth analysis of differences in age, sex, and disease stage, among others. Further research is still needed to confirm these associations and clarify the underlying mechanisms.

## Conclusion

Our review of lifestyle habits in relation to vitiligo provides several guiding suggestions. Additionally, it is recommended that both normal individuals and those with vitiligo refrain from smoking and excessive alcohol consumption. We recommend that every patient with vitiligo undergo blood tests for vitamin E, vitamin B12, zinc, and copper. If the test results indicate low levels of these nutrients, supplementation should be considered under the guidance of a doctor. However, supplementation with vitamin C, vitamin D, selenium, and folic acid may not be necessary. Furthermore, patients with vitiligo avoid high-fat diets, as these have been associated with negative health effects. Given the increased risk of sleep disorders and sexual dysfunction in individuals with vitiligo, seeking specialized help from healthcare professionals in these areas is advisable if necessary. Additionally, sun protection is crucial, particularly for women with high tanning abilities, as exposure to UV rays can have implications for the development and progression of vitiligo. Finally, regardless of the presence or absence of vitiligo, it is recommended to avoid the use of permanent hair dyes due to their potential association with an increased risk of vitiligo.

These guiding suggestions aim to provide individuals with vitiligo with lifestyle recommendations that may help manage their condition and improve their overall wellbeing. However, it is important to consult healthcare professionals for personalized advice and treatment plans based on individual needs and circumstances.

## Data Availability

The original contributions presented in the study are included in the article/**Supplementary Material**. Further inquiries can be directed to the corresponding authors.

## Supplementary material

Supplementary Figure 1 Sensitivity analysis of the serum zinc levels between patients with vitiligo and controls 95% CI, 95% confidence interval.

Supplementary Figure 2 Sensitivity analysis of the serum vitamin B12 levels between patients with vitiligo and controls 95% CI, 95% confidence interval.

Supplementary Figure 3 Sensitivity analysis of the serum copper levels between patients with vitiligo and controls 95% CI, 95% confidence interval.

Supplementary Figure 4 Sensitivity analysis of the serum selenium levels between patients with vitiligo and controls 95% CI, 95% confidence interval.

Supplementary Figure 5 Meta-analysis of smoking in patients with vitiligo and controls 95% CI, 95% confidence interval.

Supplementary Figure 6 Meta-analysis of alcohol consumption in patients with vitiligo and controls 95% CI, 95% confidence interval.

Supplementary Figure 7 Meta-analysis of the serum vitamin C levels in patients with vitiligo and controls 95% CI, 95% confidence interval.

Supplementary Figure 8 Meta-analysis of the serum vitamin B12 levels in patients with vitiligo and controls 95% CI, 95% confidence interval.

Supplementary Figure 9 Meta-analysis of the serum folic acid levels in patients with vitiligo and controls 95% CI, 95% confidence interval.

Supplementary Figure 10 Meta-analysis of the serum vitamin E levels in patients with vitiligo and controls 95% CI, 95% confidence interval.

Supplementary Figure 11 Meta-analysis of the serum zinc levels in patients with vitiligo and controls 95% CI, 95% confidence interval.

Supplementary Figure 12 Meta-analysis of the serum zinc levels in patients with generalized vitiligo and controls 95% CI, 95% confidence interval.

Supplementary Figure 13 Meta-analysis of the serum zinc levels in patients with localized vitiligo and controls 95% CI, 95% confidence interval.

Supplementary Figure 14 Meta-analysis of the serum zinc levels in female patients with vitiligo and controls 95% CI, 95% confidence interval.

Supplementary Figure 15 Meta-analysis of the serum zinc levels in male patients with vitiligo and controls 95% CI, 95% confidence interval.

Supplementary Figure 16 Meta-analysis of the serum zinc levels in progressive patients with vitiligo and controls 95% CI, 95% confidence interval.

Supplementary Figure 17 Meta-analysis of the serum zinc levels in stable patients with vitiligo and controls 95% CI, 95% confidence interval.

Supplementary Figure 18 Meta-analysis of the serum copper levels in patients with vitiligo and controls 95% CI, 95% confidence interval.

Supplementary Figure 19 Meta-analysis of the serum copper levels in progressive patients with vitiligo and controls 95% CI, 95% confidence interval.

Supplementary Figure 20 Meta-analysis of the serum copper levels in stable patients with vitiligo and controls 95% CI, 95% confidence interval.

Supplementary Figure 21 Meta-analysis of the serum copper levels in male patients with vitiligo and controls 95% CI, 95% confidence interval.

Supplementary Figure 22 Meta-analysis of the serum copper levels in female patients with vitiligo and controls 95% CI, 95% confidence interval.

Supplementary Figure 23 Meta-analysis of the serum selenium levels in patients with vitiligo and controls 95% CI, 95% confidence interval.

Supplementary Figure 24 Meta-analysis of the serum selenium levels in progressive patients with vitiligo and controls 95% CI, 95% confidence interval.

Supplementary Figure 25 Meta-analysis of the serum selenium levels in stable patients with vitiligo and controls 95% CI, 95% confidence interval.

Supplementary Figure 26 Meta-analysis of the serum iron levels in patients with vitiligo and controls 95% CI, 95% confidence interval.

Supplementary Table 2 Search strategy.
