# Supplementary material for: Healthy lifestyle choices: new insights into vitiligo management
Source: Front Immunol. 2024 Nov 18;15:1440705. doi: 10.3389/fimmu.2024.1440705 (PMC11609173; doi:10.3389/fimmu.2024.1440705)

Fig S1. Meta-analysis of serum zinc levels in patients with vitiligo and controls.

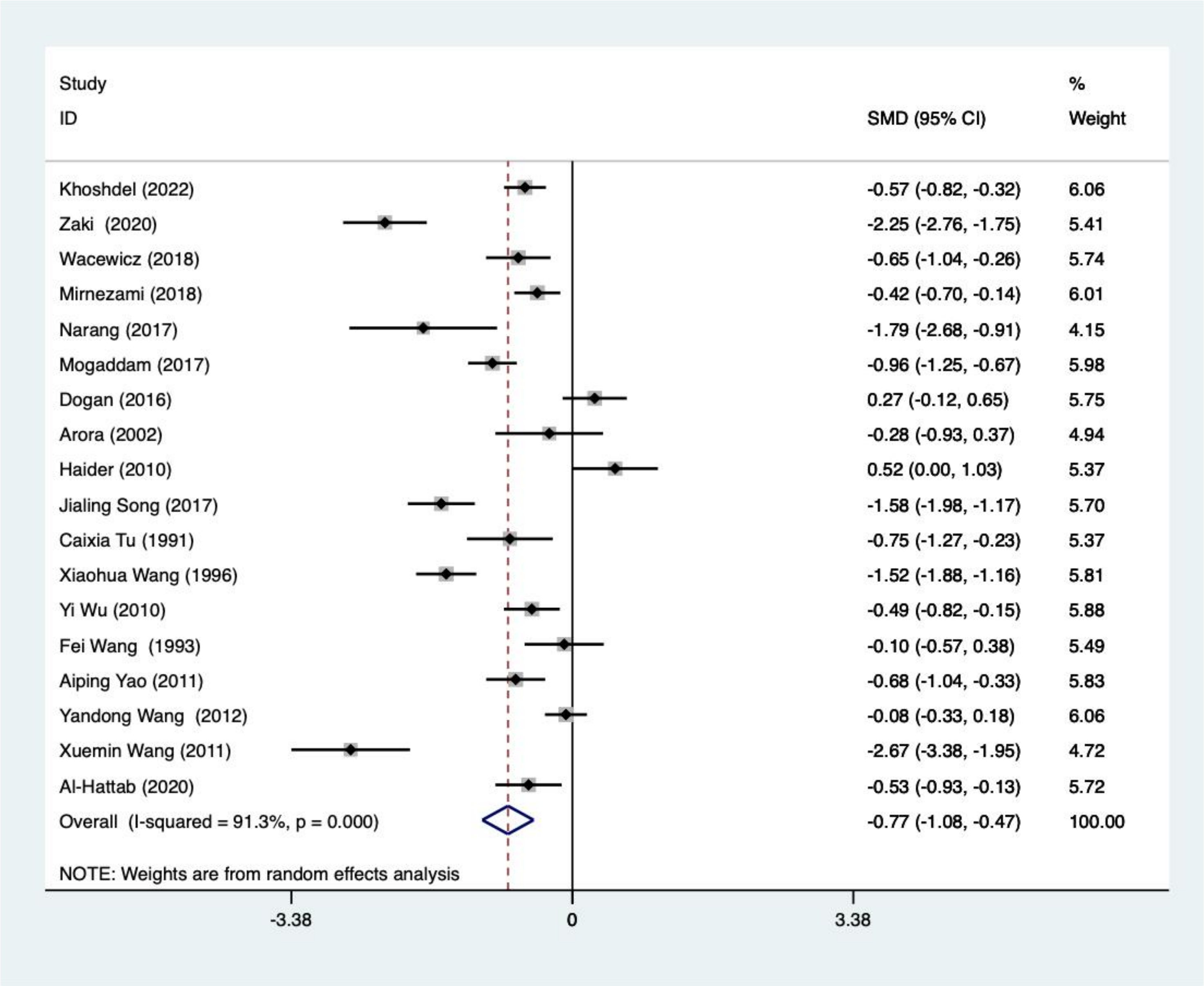

Fig S2. Meta-analysis of serum zinc levels in patients with generalized vitiligo and control group.

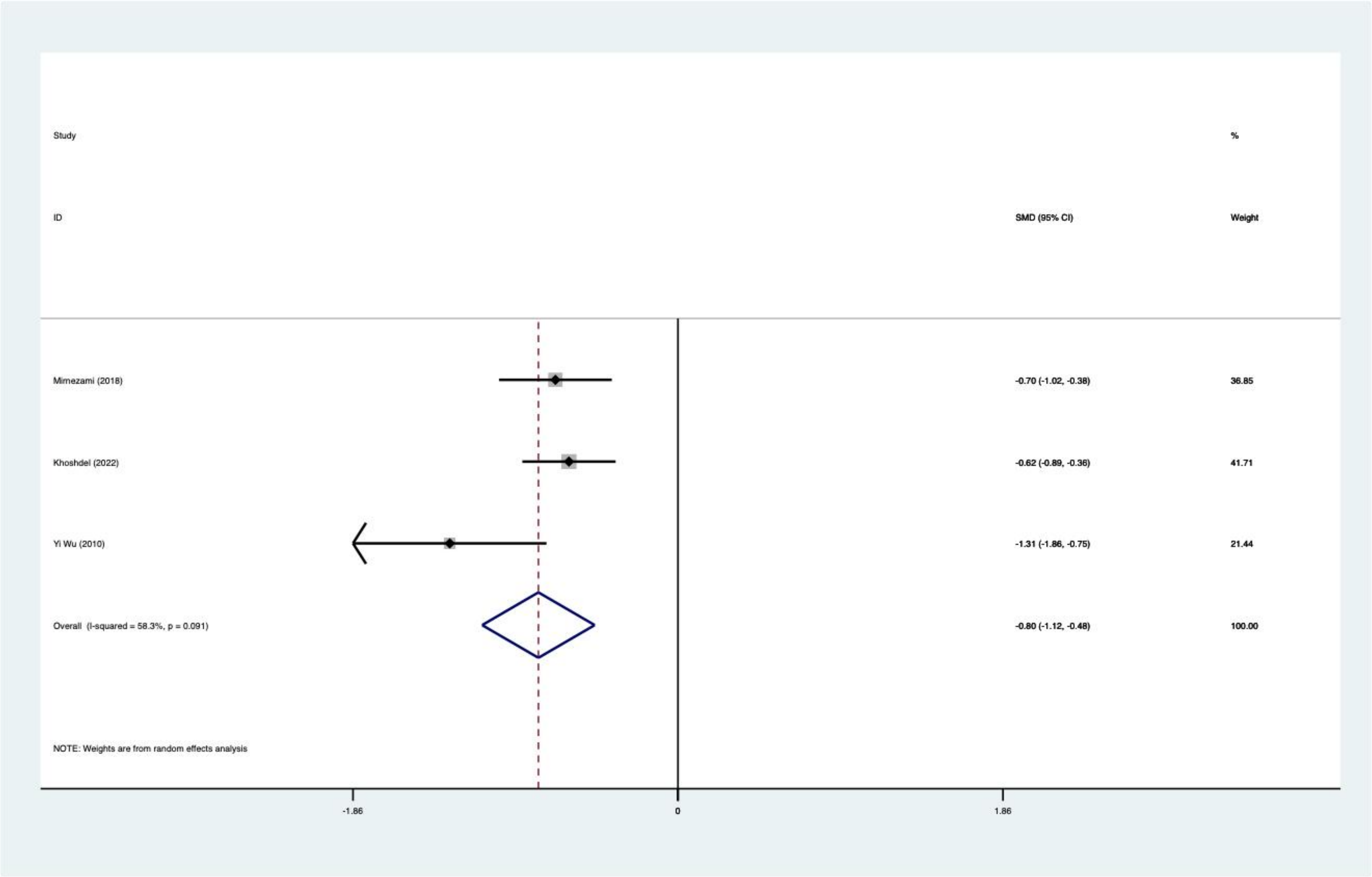

Fig S3. Meta-analysis of serum zinc levels in patients with Localized vitiligo and control group.

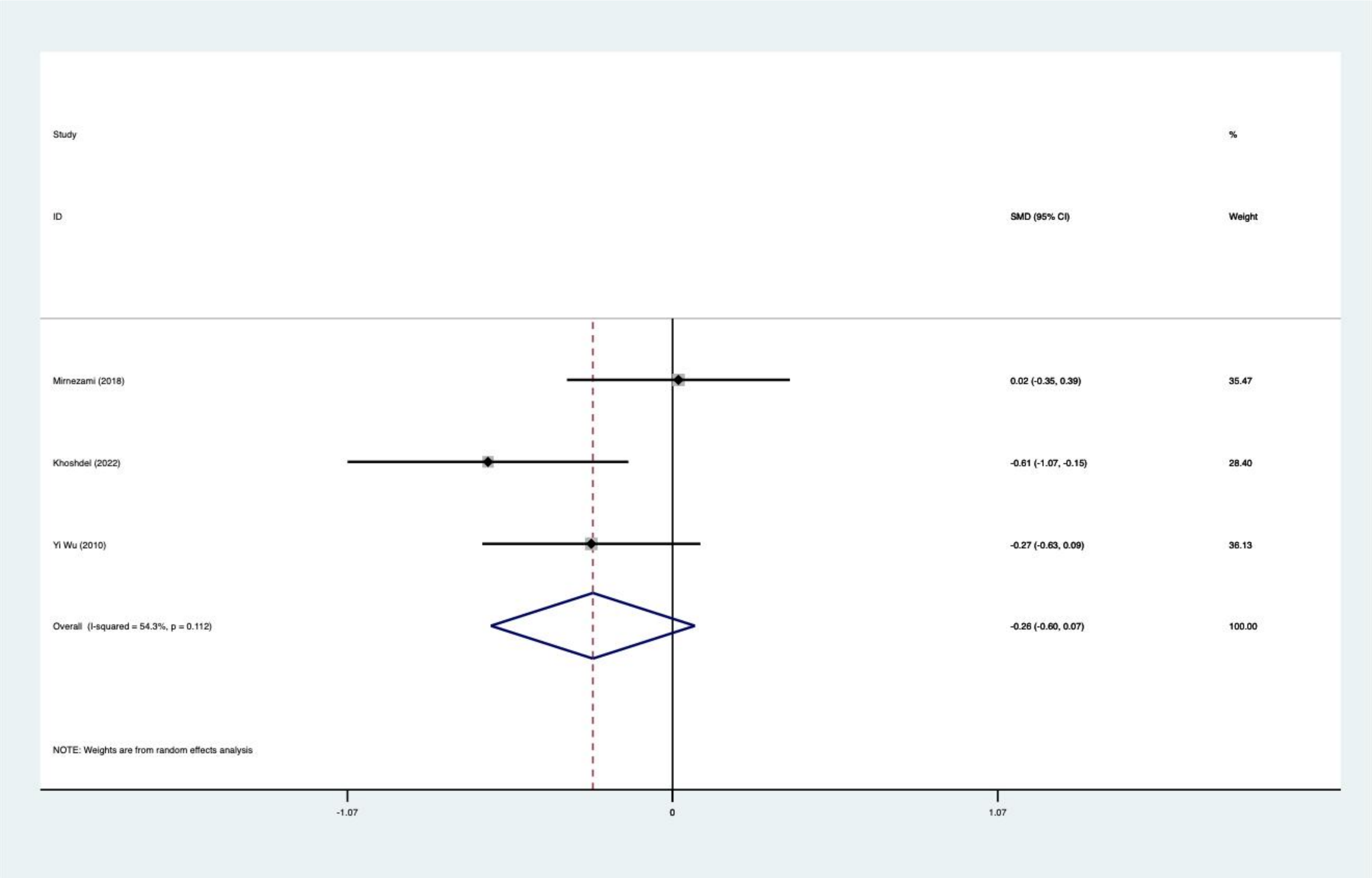

Fig S4. Meta-analysis of serum zinc levels in female patients with vitiligo and control group.

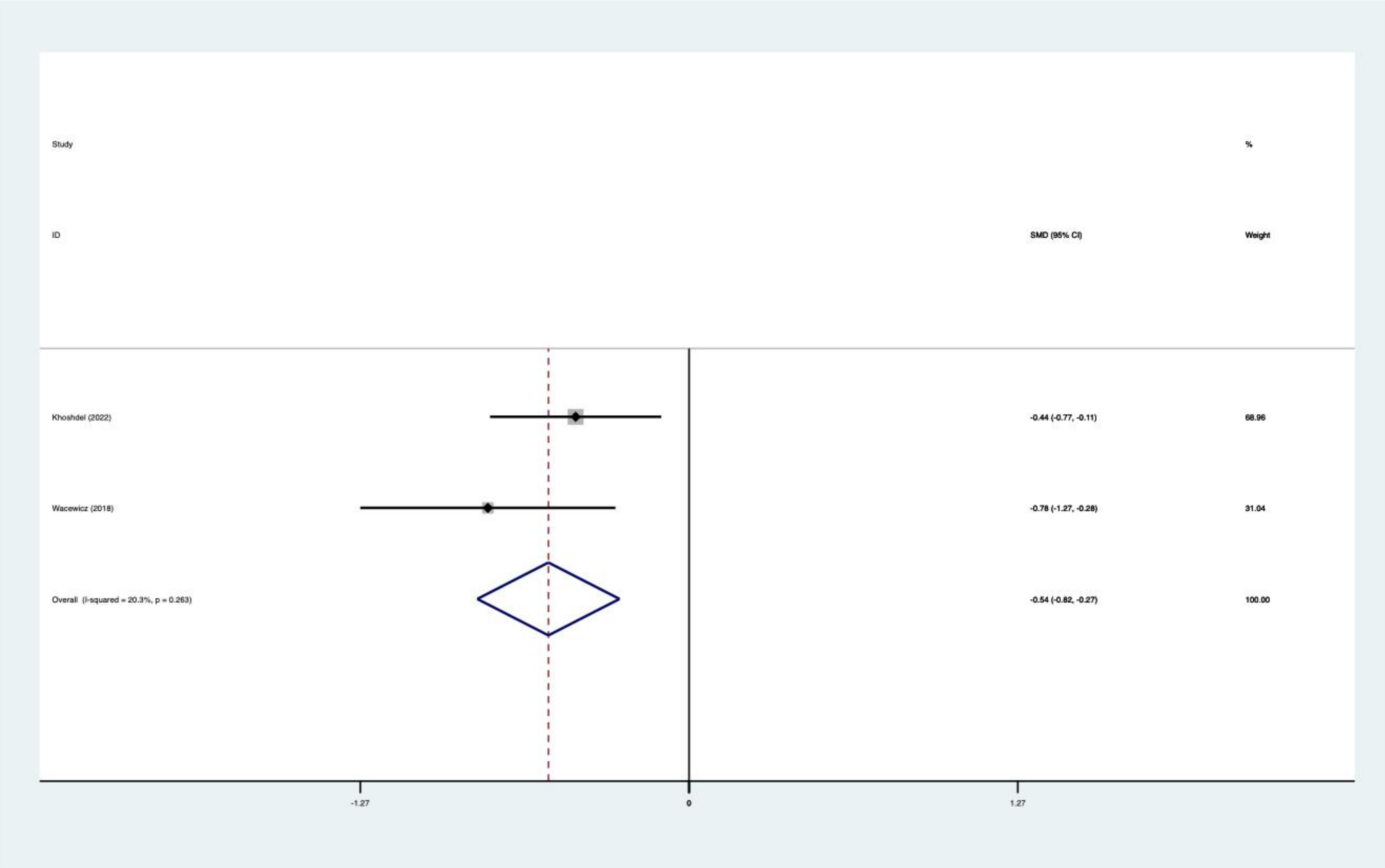

Fig S5. Meta-analysis of serum zinc levels in male patients with vitiligo and control group.

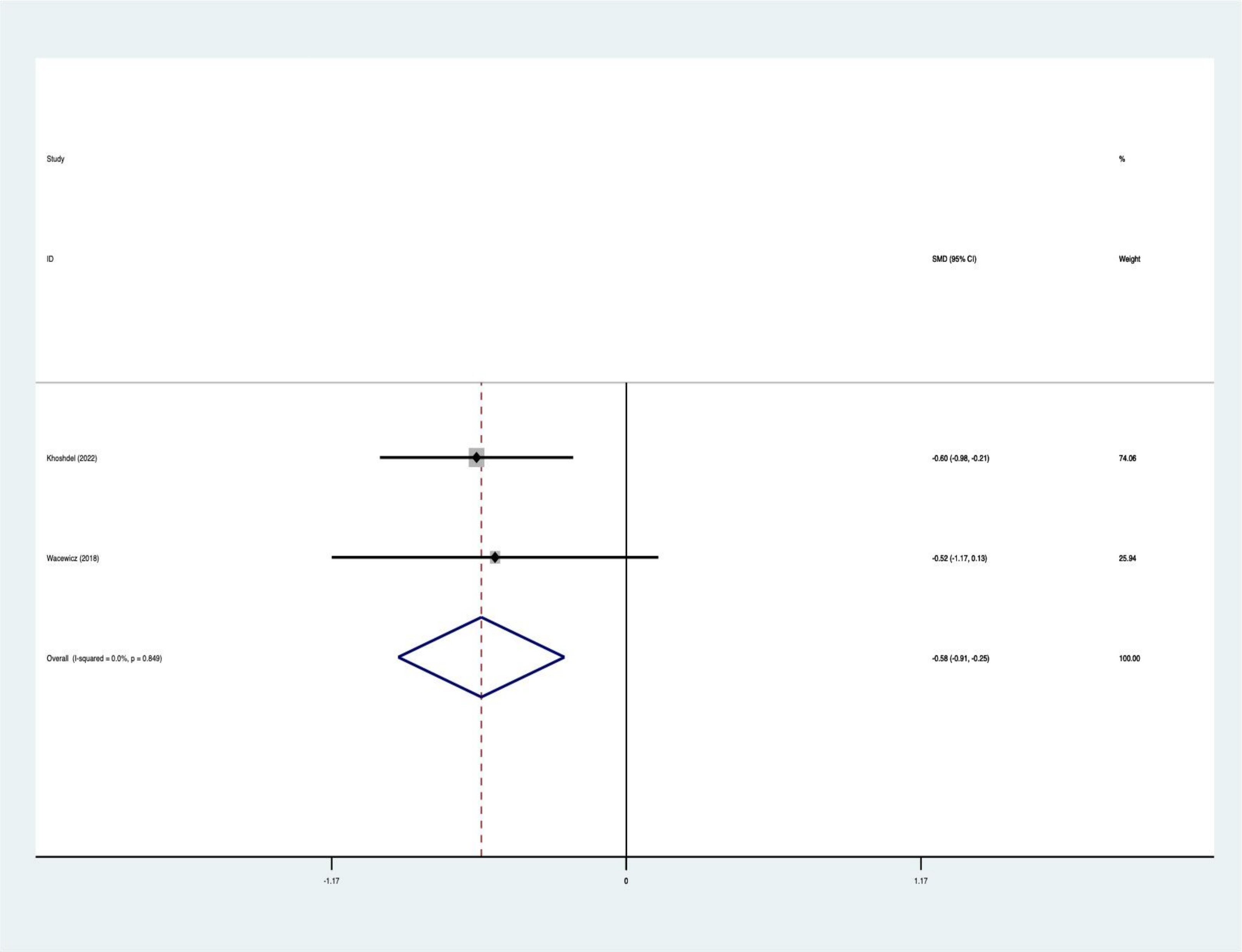

Fig S6. Meta-analysis of serum zinc levels in progressive patients with vitiligo and control group.

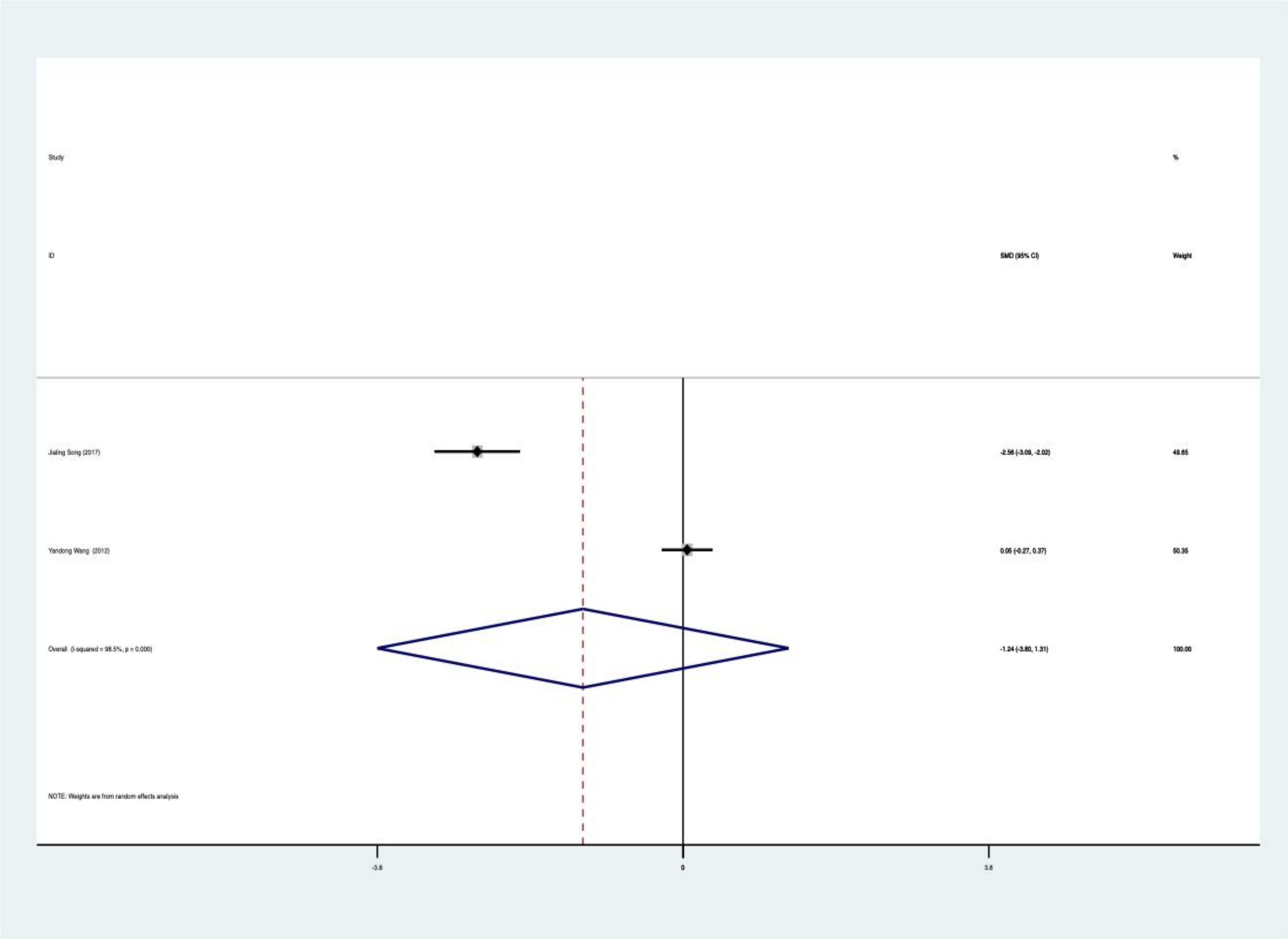

Fig S7. Meta-analysis of serum zinc levels in stable patients with vitiligo and control group.

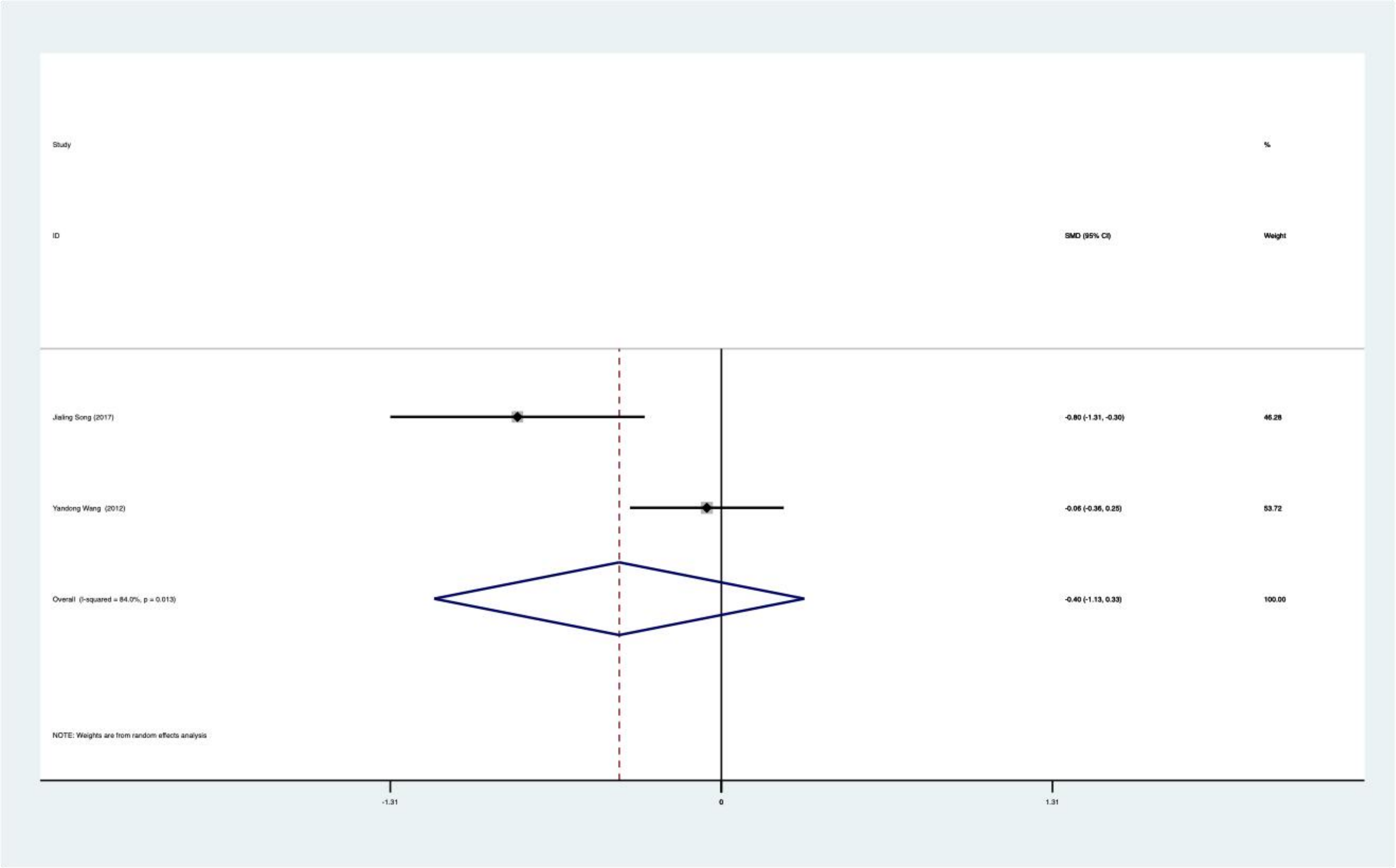

Fig S8. Meta-analysis of serum Vit B12 levels in patients with vitiligo and controls.

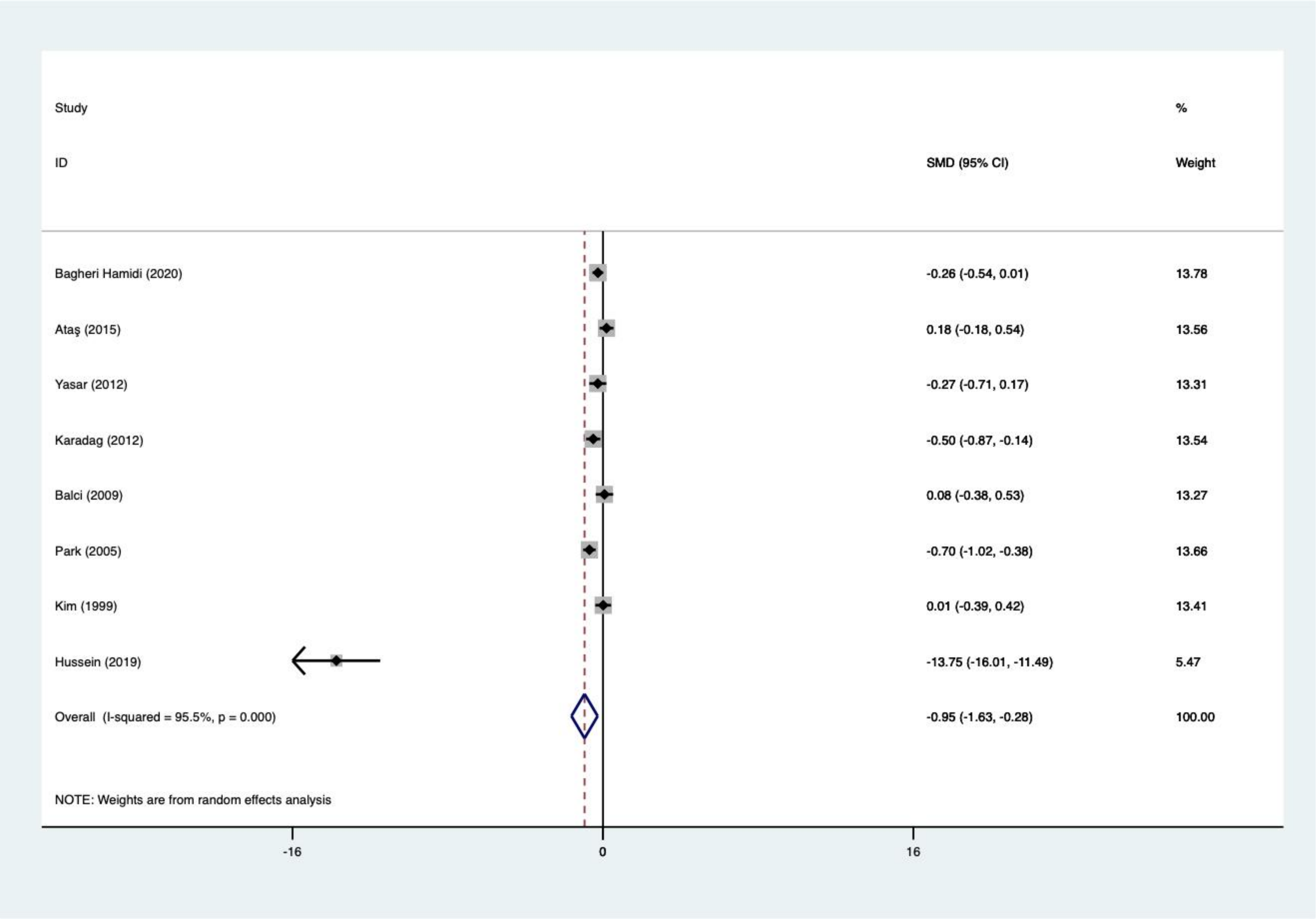

Fig S9. Meta-analysis of serum Folic acid levels in patients with vitiligo and controls.

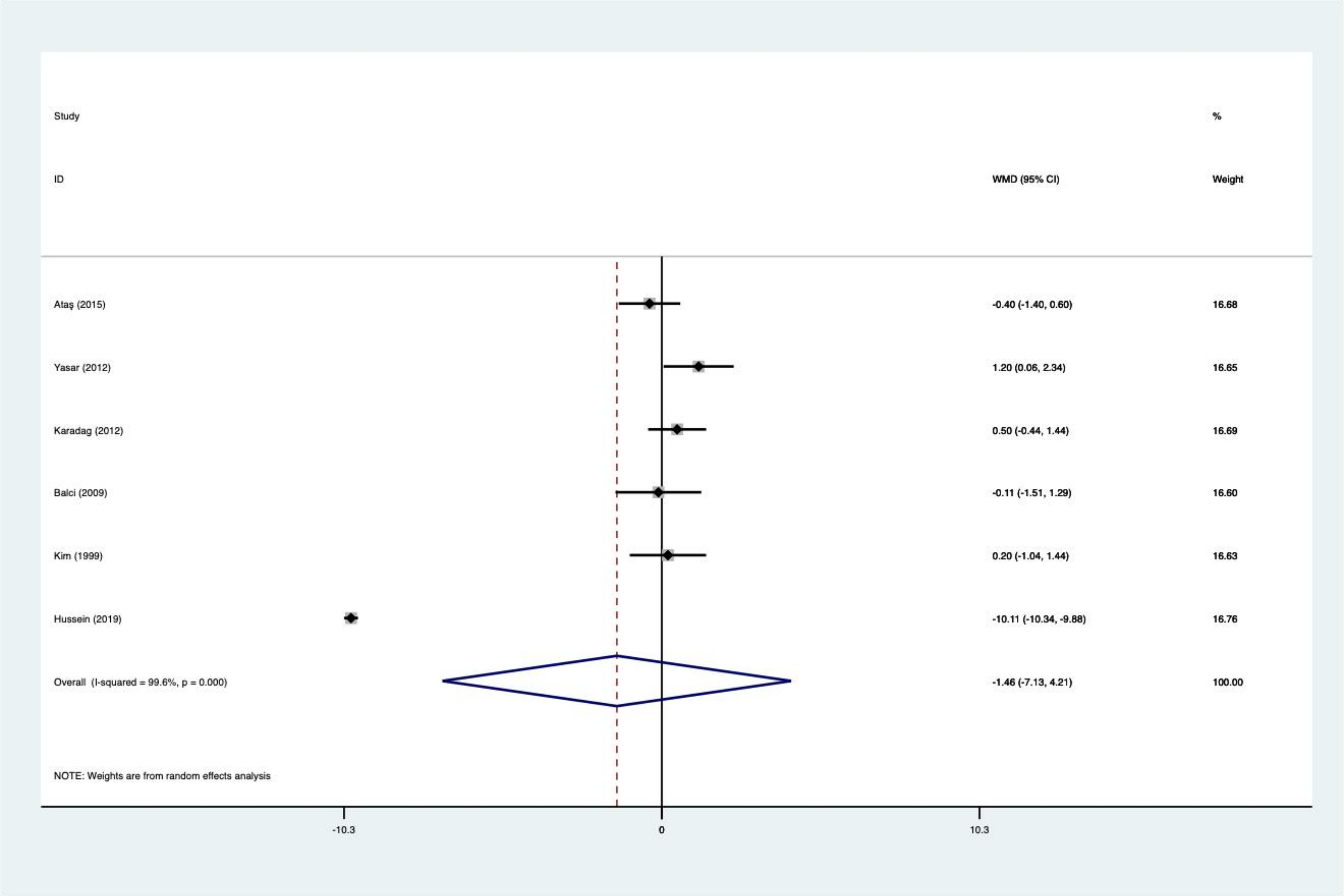

Fig S10. Meta-analysis of serum Vit E levels in patients with vitiligo and controls.

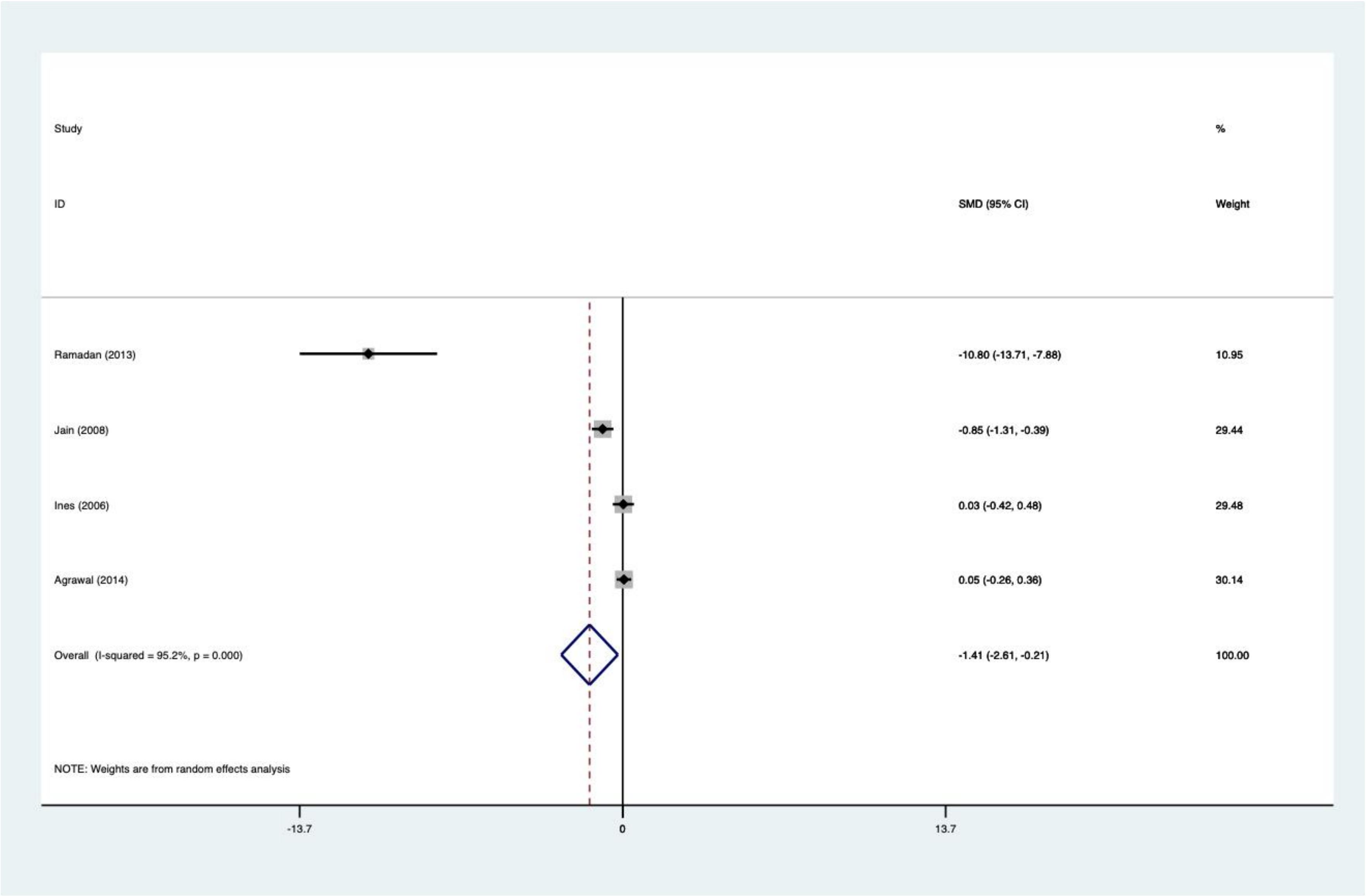

Fig S11. Meta-analysis of serum Vit C levels in patients with vitiligo and controls.

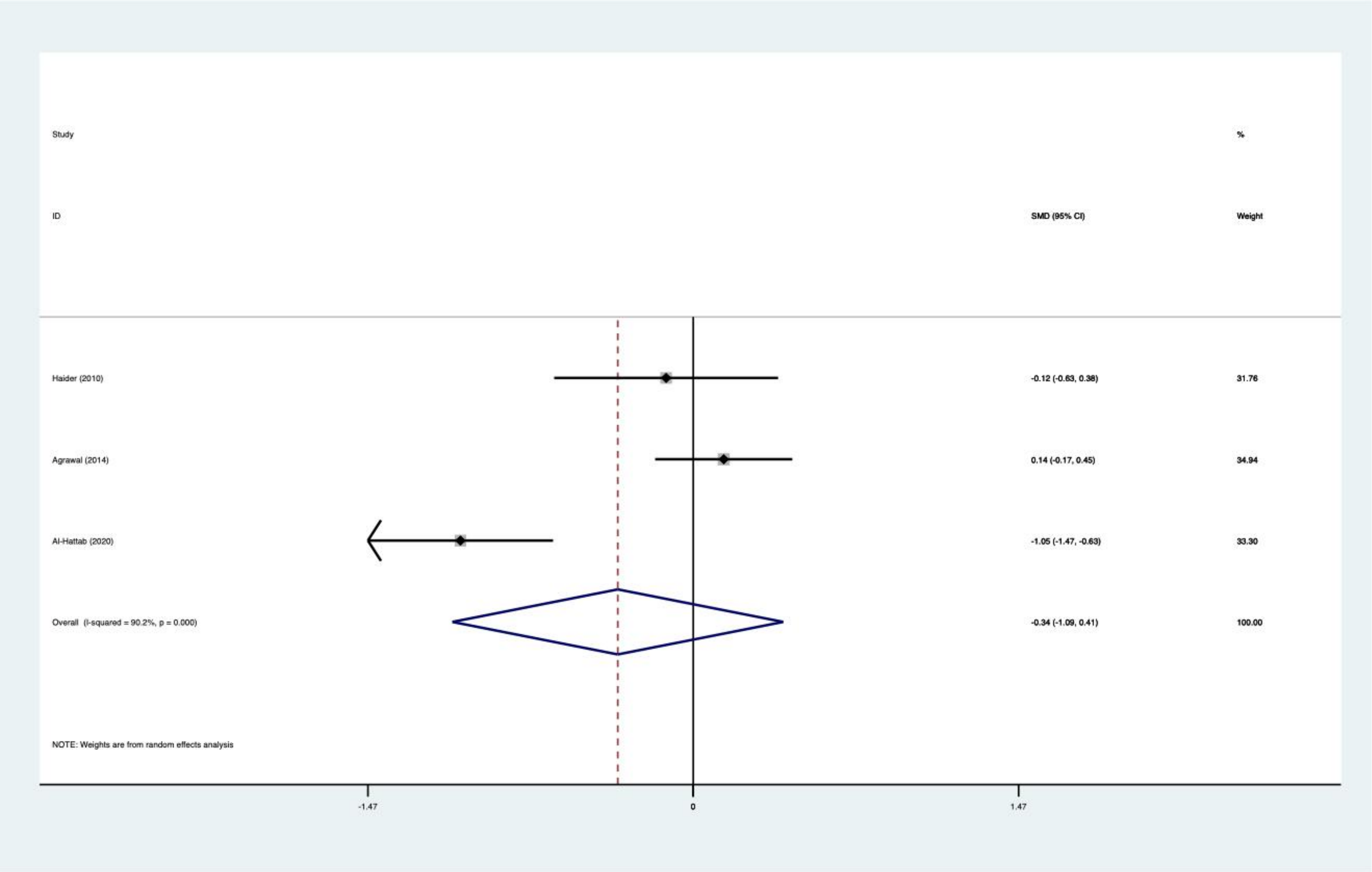

Fig S12. Meta-analysis of serum copper levels in patients with vitiligo and controls.

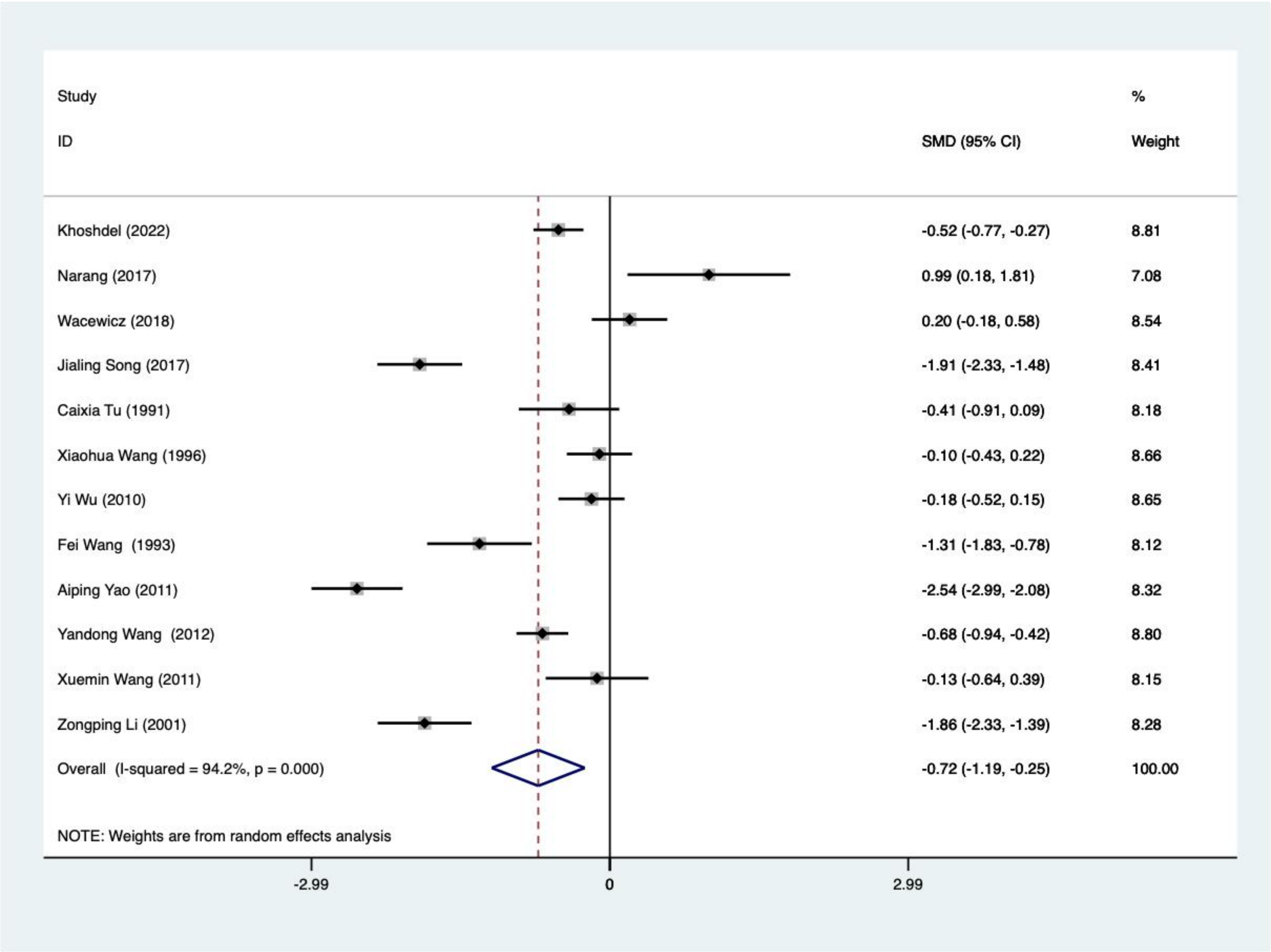

Fig S13. Meta-analysis of serum copper levels in progressive patients with vitiligo and controls.

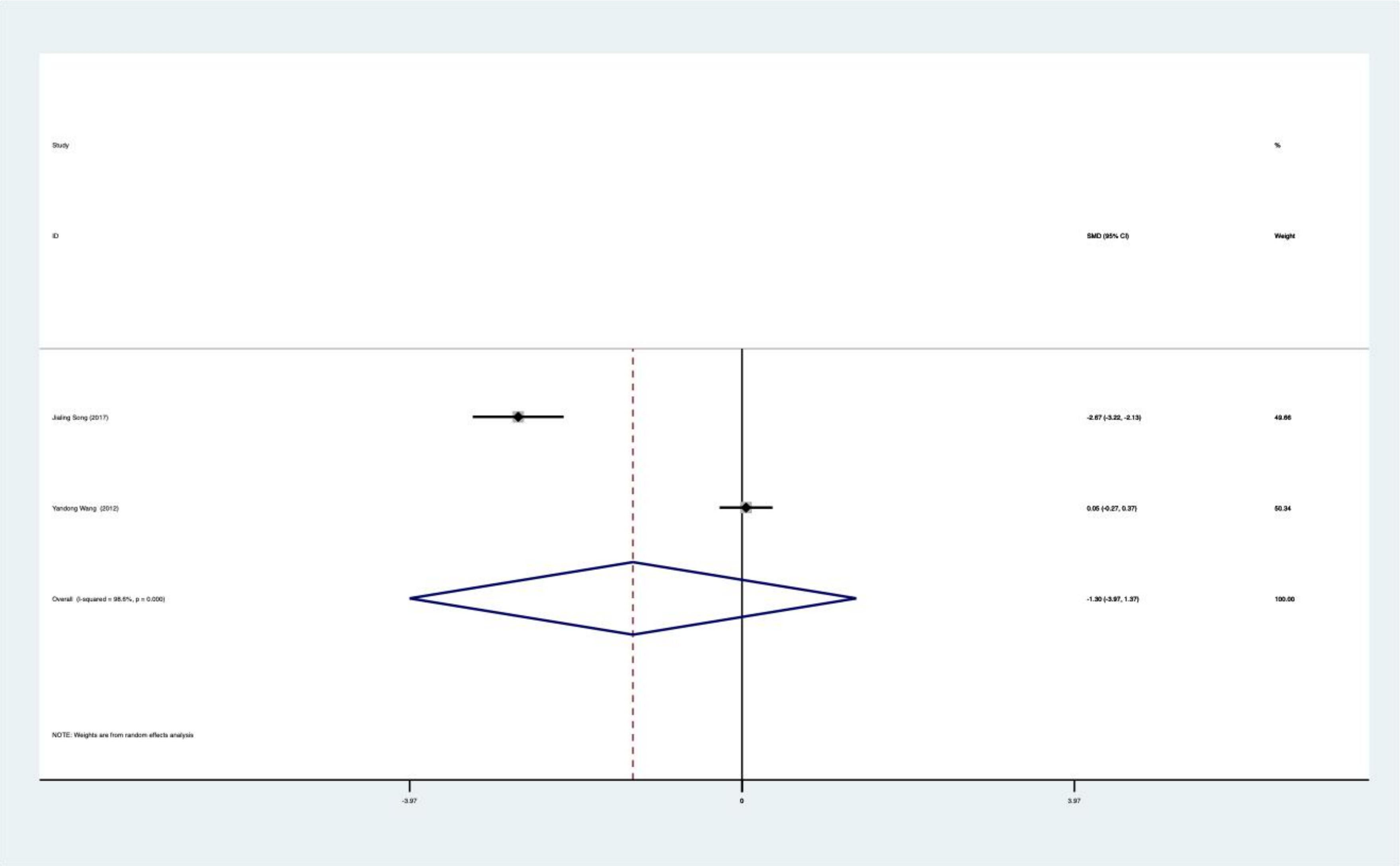

Fig S14. Meta-analysis of serum copper levels in stable patients with vitiligo and controls.

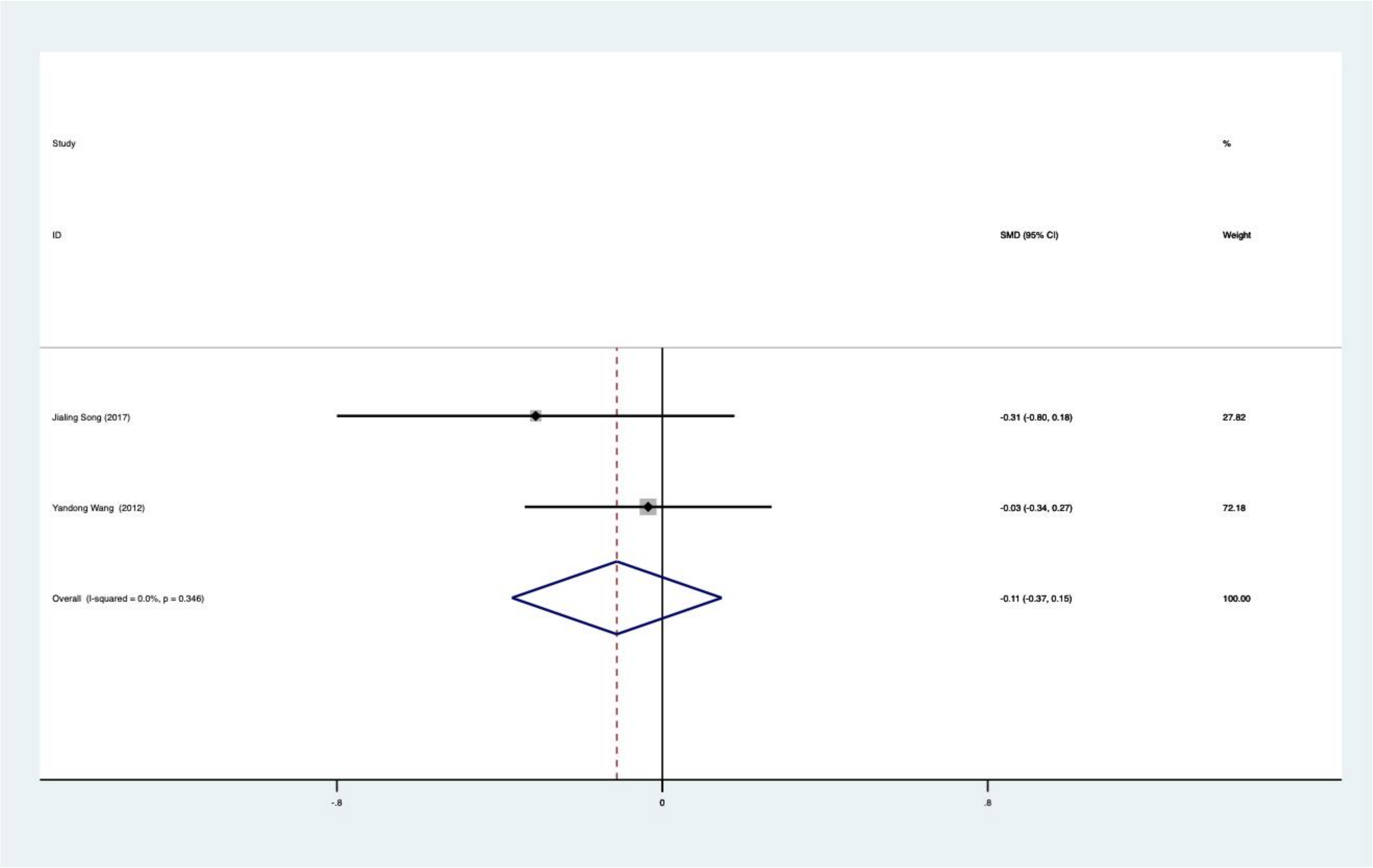

Fig S15. Meta-analysis of serum copper levels in female patients with vitiligo and controls.

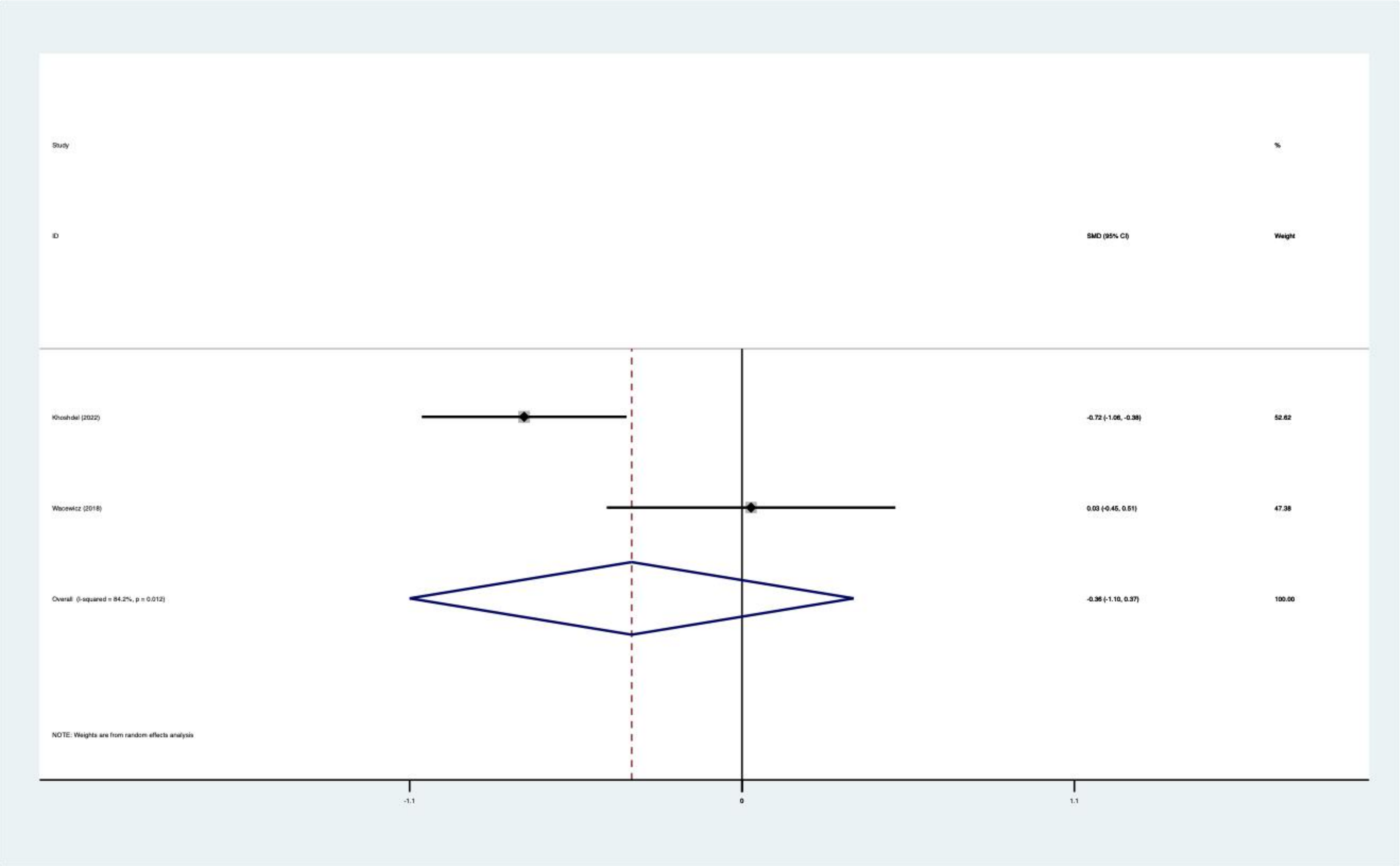

Fig S16. Meta-analysis of serum copper levels in male patients with vitiligo and controls.

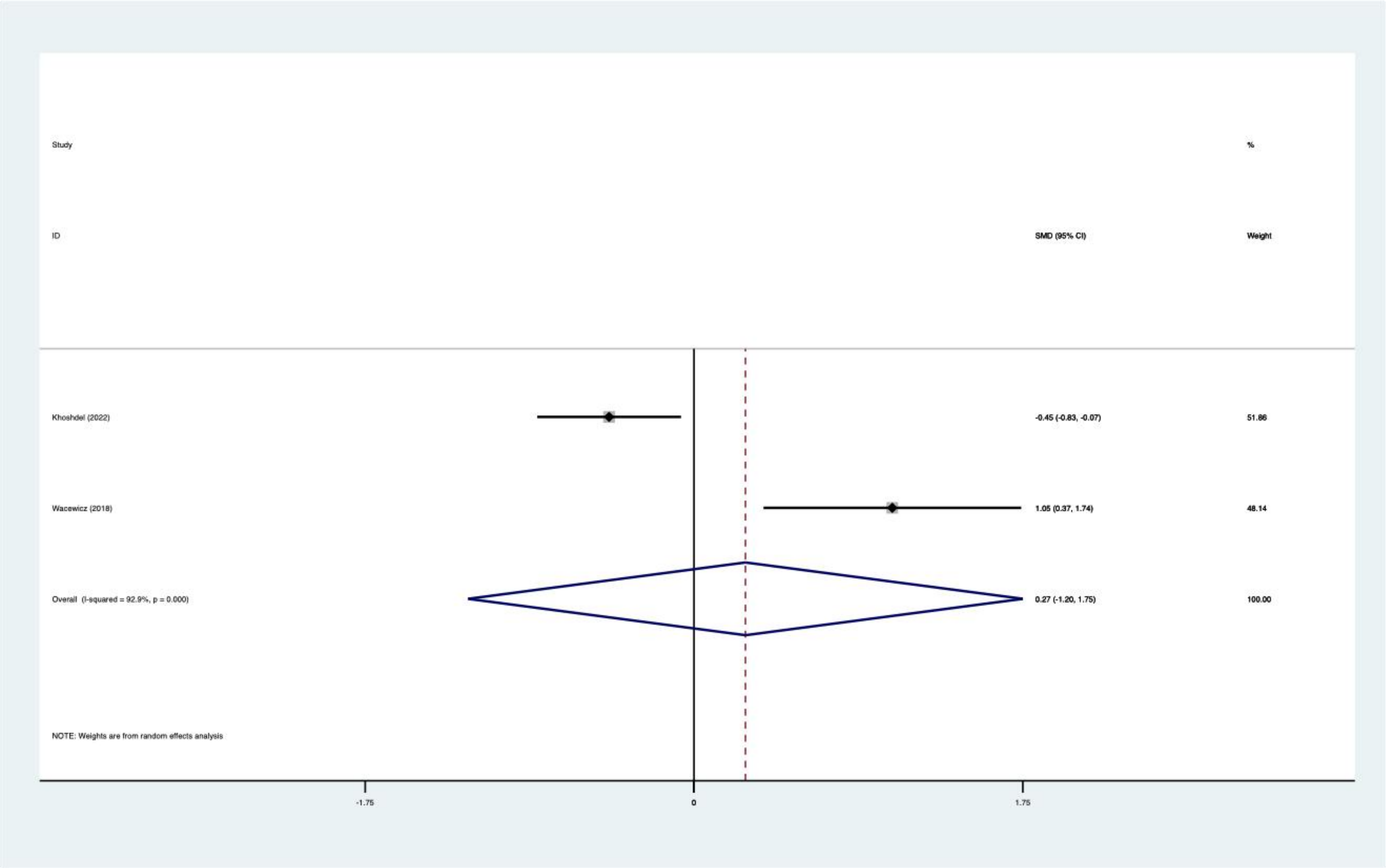

Fig S17. Meta-analysis of serum selenium levels in patients with vitiligo and controls.

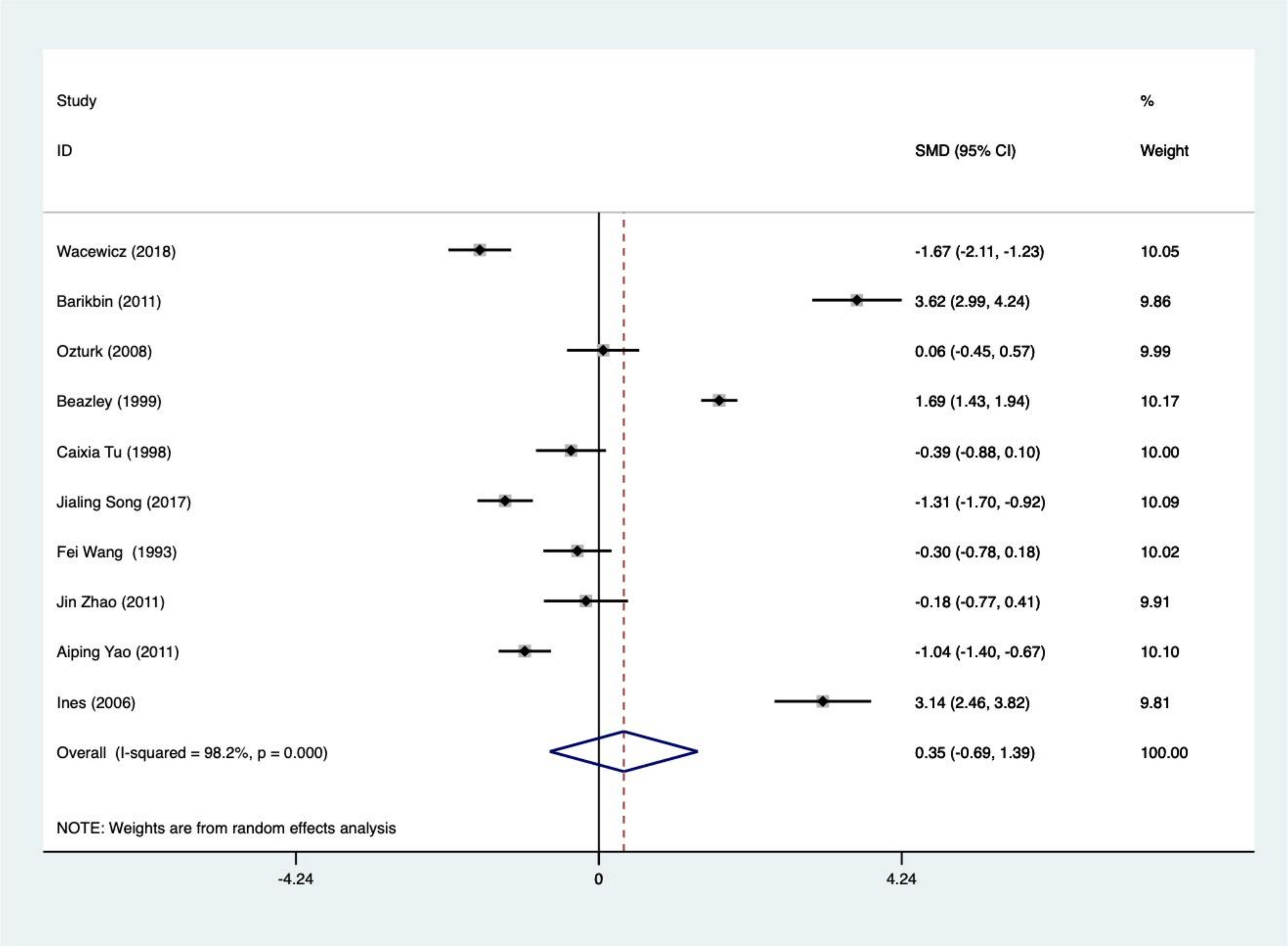

Fig S18. Meta-analysis of serum selenium levels in progressive patients with vitiligo and controls.

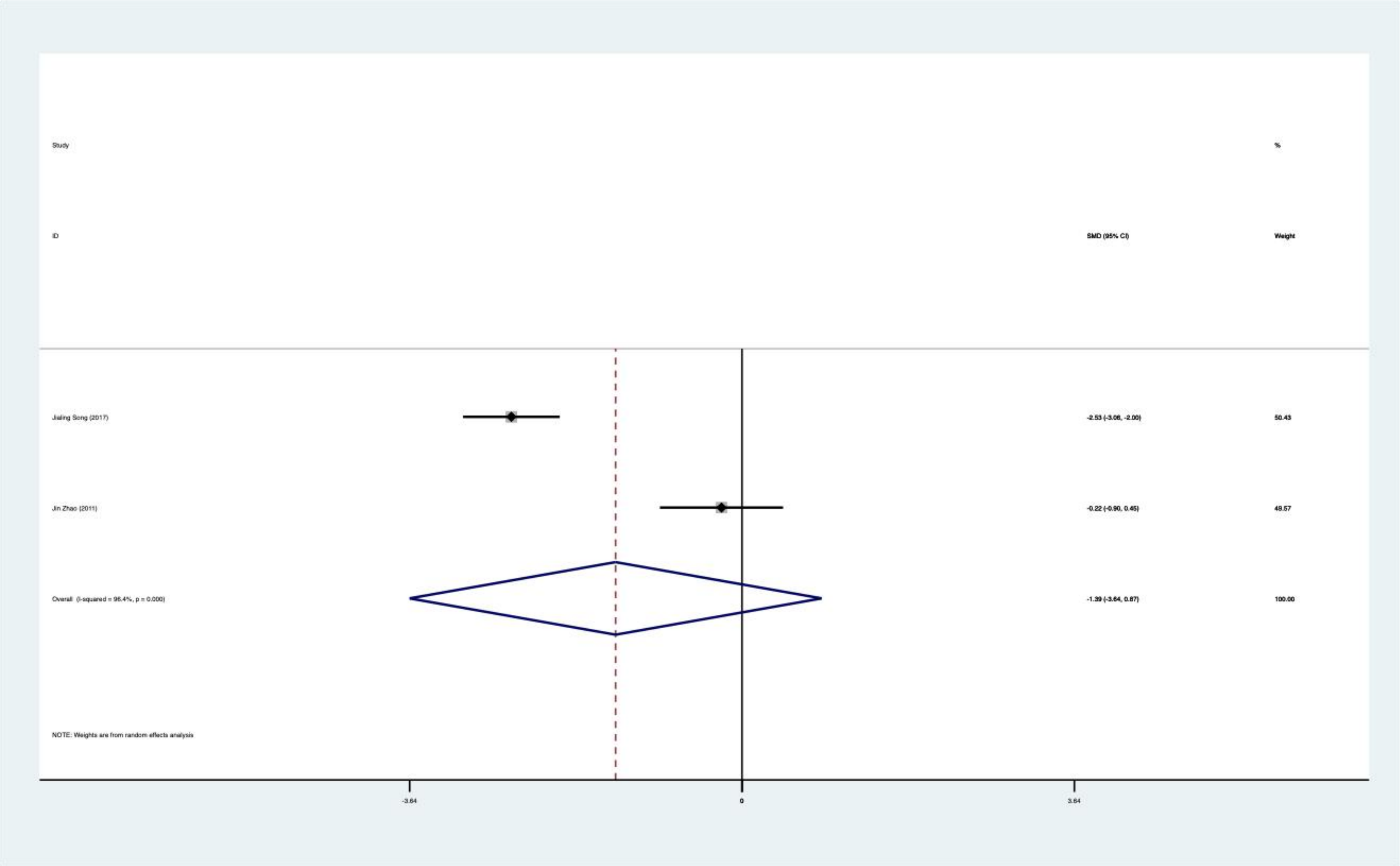

Fig S19. Meta-analysis of serum selenium levels in stable patients with vitiligo and controls.

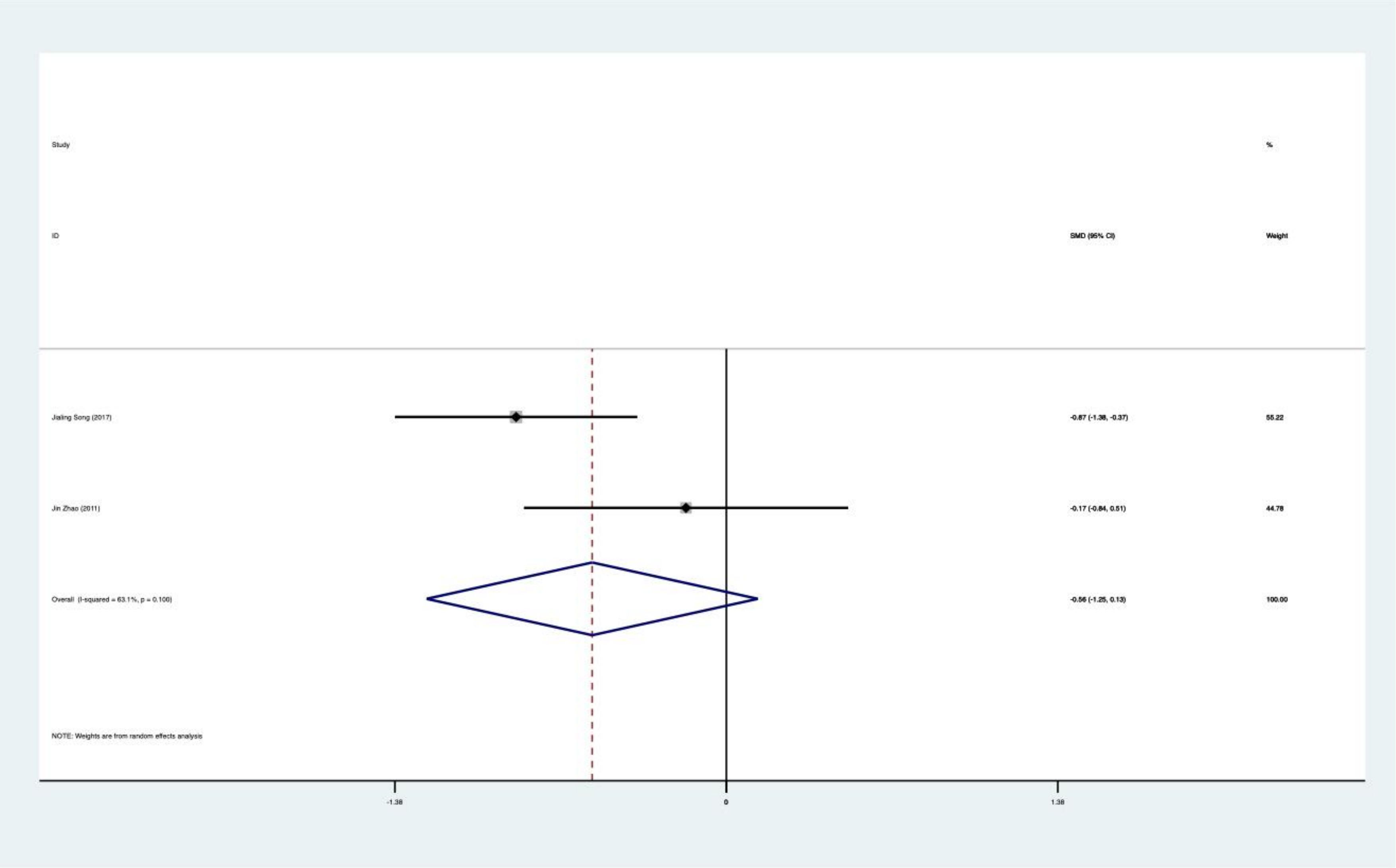

Fig S20. Meta-analysis of serum iron levels in patients with vitiligo and controls.

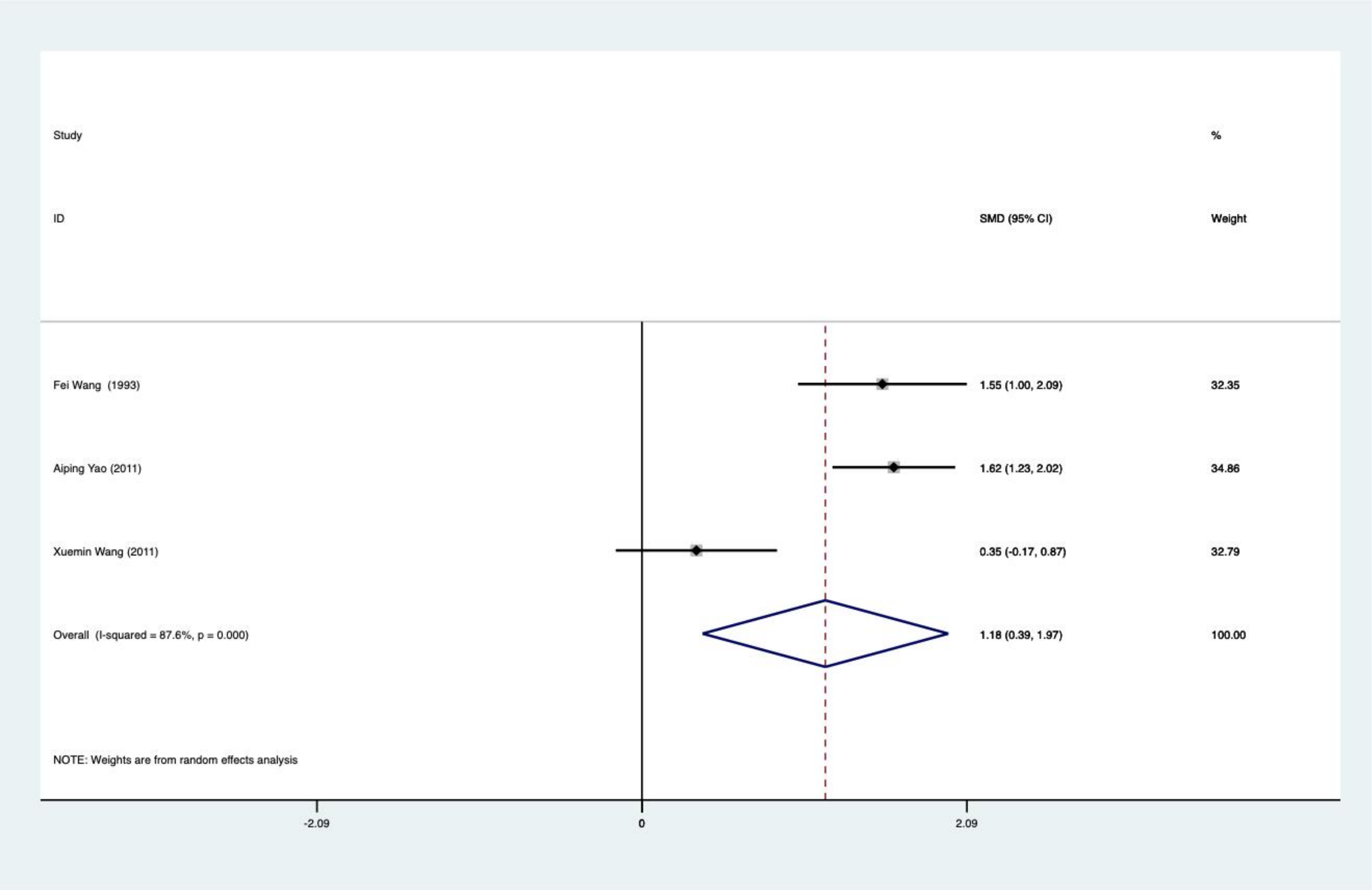

Fig S21. Meta-analysis of smoking in patients with vitiligo and controls.

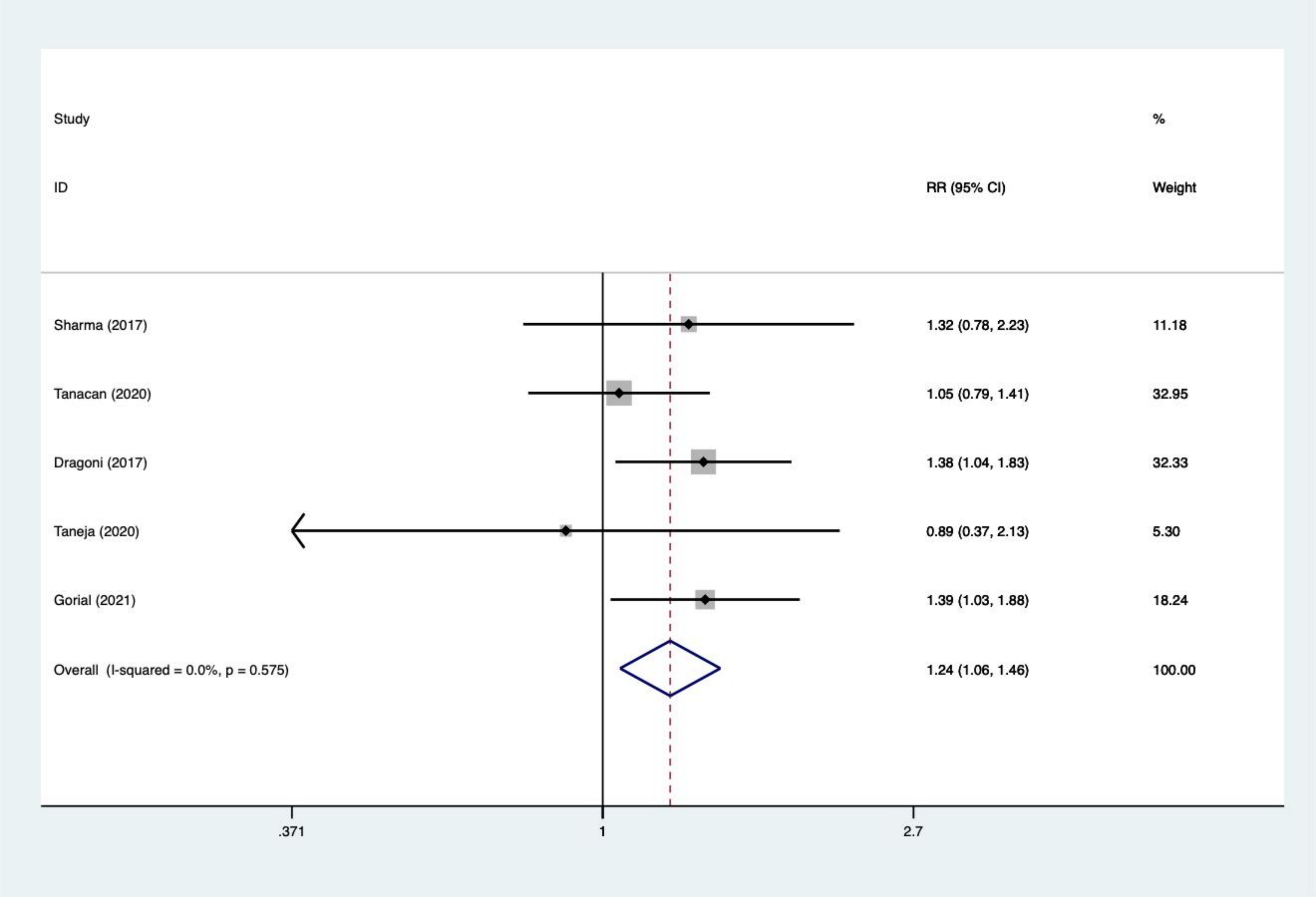

Fig S22. Meta-analysis of alcohol consumption in patients with vitiligo and controls.

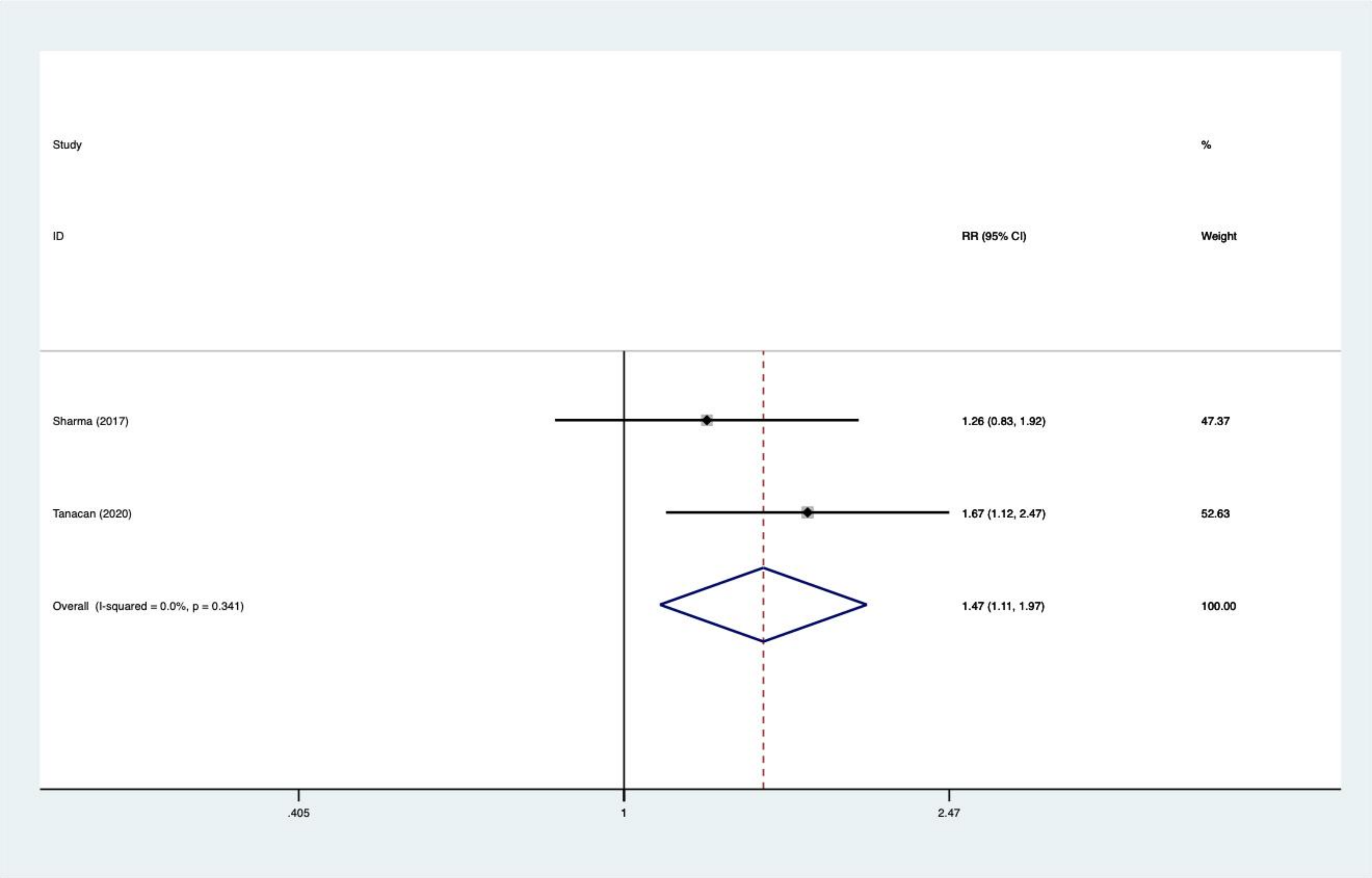

Fig S23. Sensitivity analysis of serum zinc levels between vitiligo patients and controls.

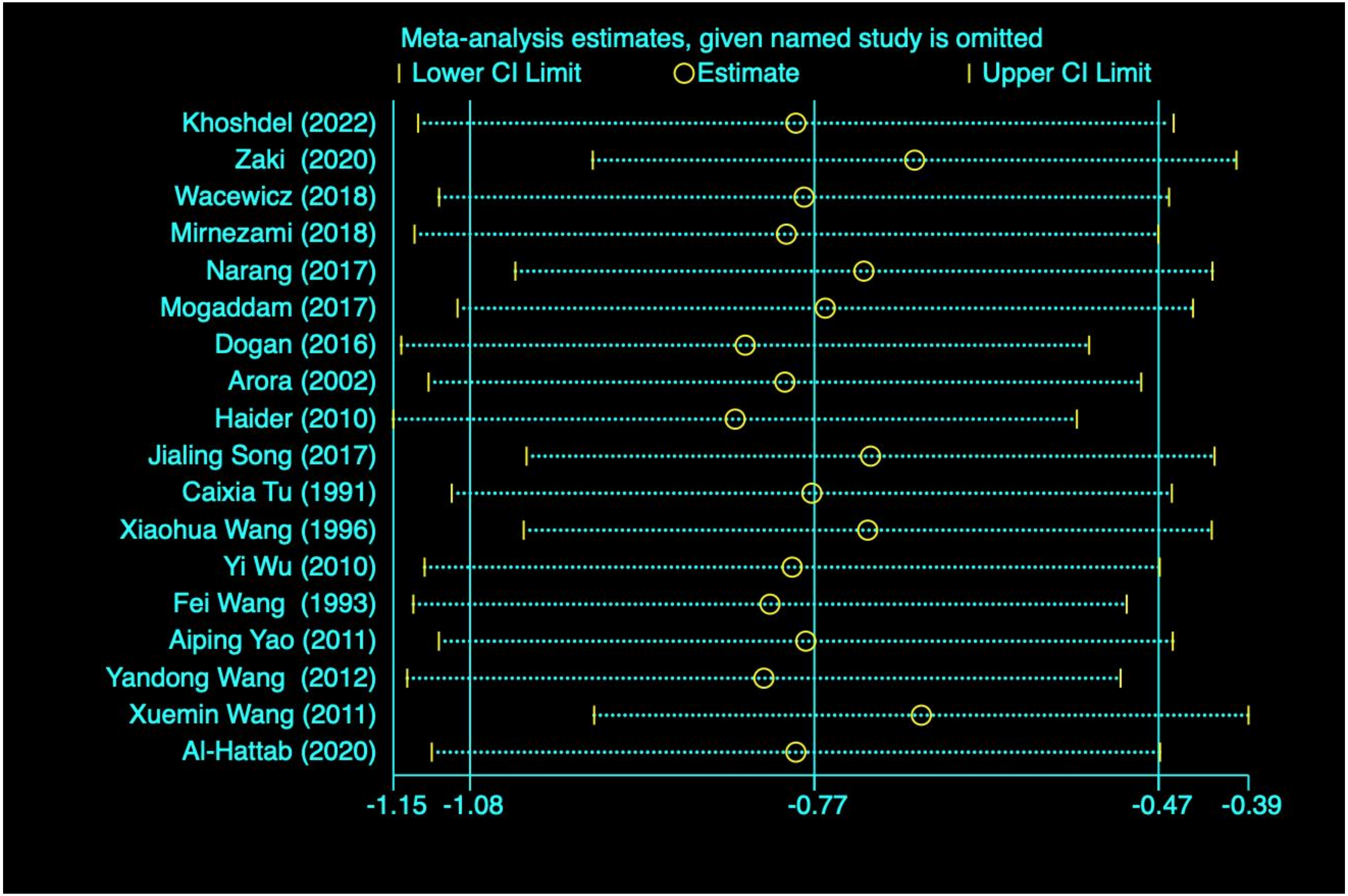

Fig S24. Sensitivity analysis of serum Vit B12 levels between vitiligo patients and controls.

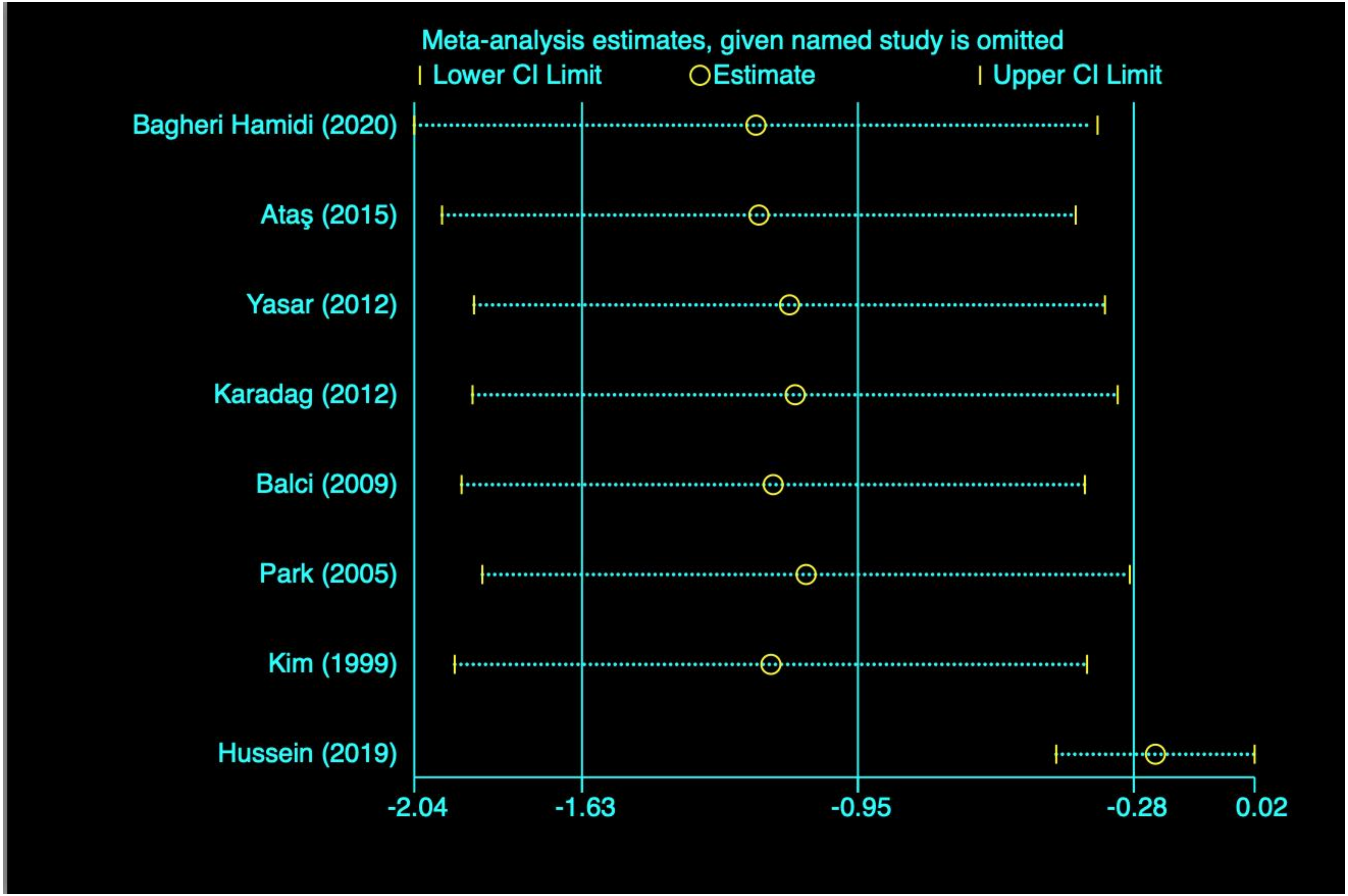

Fig S25. Sensitivity analysis of serum copper levels between vitiligo patients and controls.

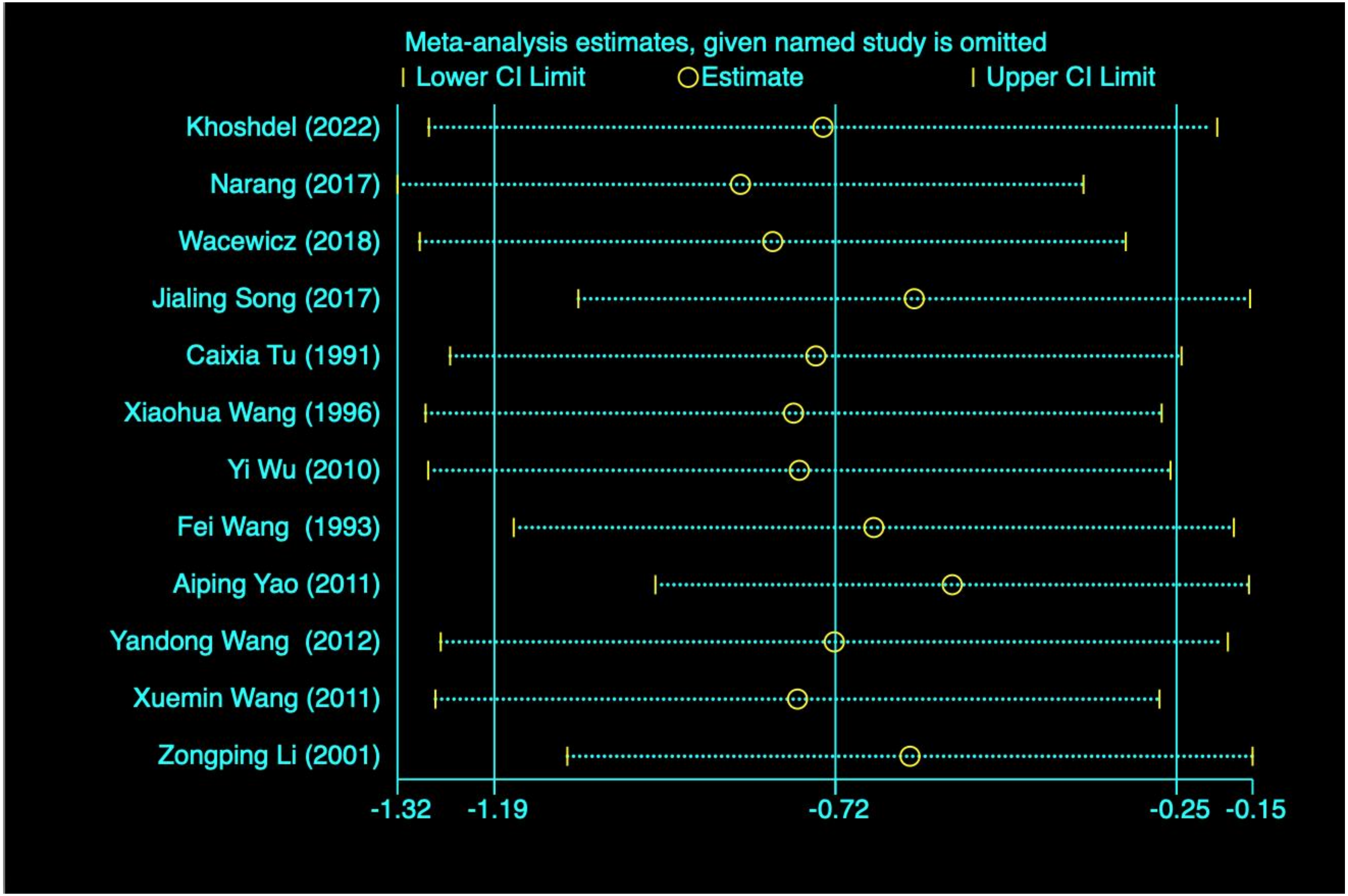

Fig S26. Sensitivity analysis of serum selenium levels between vitiligo patients and controls.

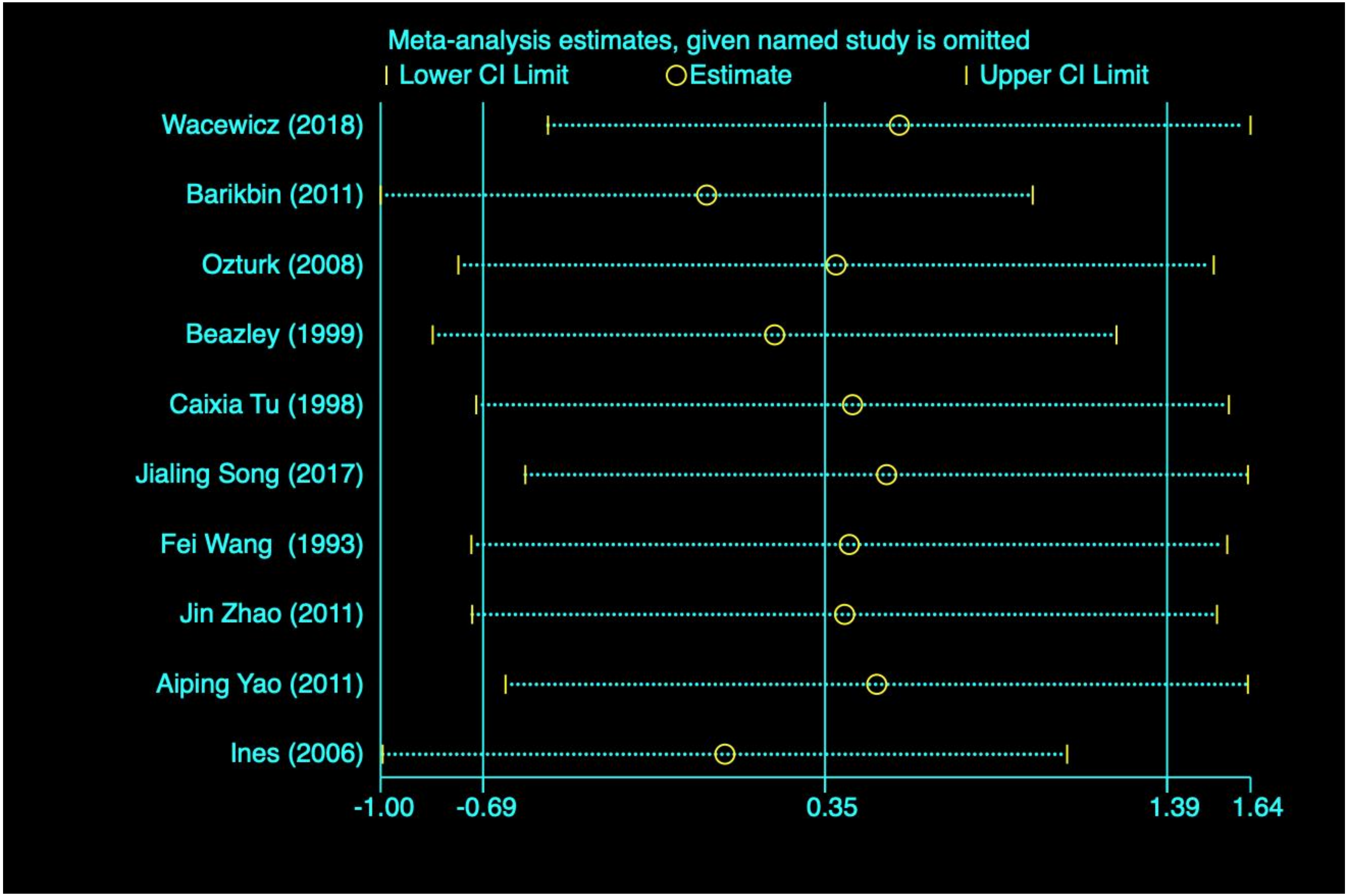

Supplement: Supplementary Figure 1 — Sensitivity analysis of the serum zinc levels between patients with vitiligo and controls 95% CI, 95% confidence interval. [file DataSheet1.pdf]
