# Supplementary material for: Healthy lifestyle choices: new insights into vitiligo management
Source: Front Immunol. 2024 Nov 18;15:1440705. doi: 10.3389/fimmu.2024.1440705 (PMC11609173; doi:10.3389/fimmu.2024.1440705)
Supplement: Supplementary file 2 [file Table1.doc]

**MOOSE Checklist**

**Healthy lifestyle choices for vitiligo: new insights into vitiligo management**

Xin Liang^a¶^, Fei Guo^b¶^, Qian Fan^a^, Xiaoce Cai^b^, Jiao Wang^b^, Jiale Chen^b^, Fang Liu^a^, Yuhua Du^a^, Yan Chen^a,*^, and Xin Li^a,b,c,*^

^a^Chinese Medicine Department, Songnan Town Community Health Service Center, Baoshan District, Shanghai 200441, China

^b^Department of Dermatology, Yueyang Hospital of Integrated Traditional Chinese and Western Medicine, Shanghai University of Traditional Chinese Medicine, Shanghai 200437, China

^c^Institute of Dermatology, Shanghai Academy of Traditional Chinese Medicine, Shanghai 201203, China

^¶^These authors have contributed equally to this work.

**Corresponding authors:**

Xin Li

Department of Dermatology, Songnan Town Community Health Service Center, Yueyang Hospital of Integrated Traditional Chinese and Western Medicine, Shanghai University of Traditional Chinese Medicine, Shanghai 200437, China

Phone: +86 13661956326

Fax: +86 021-65162629

Email: [13661956326@163.com](mailto:13661956326@163.com)

Yan Chen

Chinese Medicine Department, Songnan Town Community Health Service Center, Shanghai 200441, China

Phone: +86 13916947368

Fax: +86 021-56466314

^Email^: [snyygh@163.com](mailto:snyygh@163.com)

| **Criteria** | | **Brief description of how the criteria were handled in the meta-analysis** |
| --- | --- | --- |
| **Reporting of background should include** | |  |
|  | Problem definition | The treatment of vitiligo is complex, and providing guidance based on lifestyle habits is a good option that has not been summarized or analyzed. |
|  | Hypothesis statement | Healthy lifestyle choices for vitiligo patients |
|  | Description of study outcomes | vitiligo |
|  | Type of exposure or intervention used | Vitiligo |
|  | Type of study designs used | Observational research including cohort studies, case control studies, cross-sectional studies and Randomized controlled trials were used to analysis association between vitiligo and lifestyle all researchers. |
|  | Study population | We placed no restriction. |
| **Reporting of search strategy should include** | |  |
|  | Qualifications of searchers | The credentials of the three investigators L.Xin, G.Fei and L.Xin are indicated in the author list. |
|  | Search strategy, including time period included in the synthesis and keywords | Time period: all enrolled datebases from 1980 to December 2022.  Keywords: vitiligo; lifestyle; systematic review; meta-analysis. |
|  | Databases and registries searched | PubMed, Embase, and Cochrane Central Register databases |
|  | Search software used, name and version, including special features | We used EndNote 20 to merge retrieved citations and eliminate duplications. |
|  | Use of hand searching | The bibliographies of all retrieved studies were examined for further relevant studies. |
|  | List of citations located and those excluded, including justifications | Details of the literature search process are outlined in the flow diagram. The citation list is available upon request. |
|  | Method of addressing articles published in languages other than English | The studies we searched contained English and Chinese articles, but the ultimately articles which enrolled according to the inclusion criteria were English articles. |
|  | Method of handling abstracts and unpublished studies | We did not contacted authors for abstracts and unpublished studies on the associations of psoriasis with dementia. |
|  | Description of any contact with authors | Contact was not made with authors of the studies, as adequate information for the performance of this review was available from studies and abstracts. |
| **Reporting of methods should include** | |  |
|  | Description of relevance or appropriateness of studies assembled for assessing the hypothesis to be tested | Detailed inclusion and exclusion criteria were described in the study design section. |
|  | Rationale for the selection and coding of data | Data we analysed from each of the literatures were associated with the demographic characteristics, study design, exposure and outcome. |
|  | Assessment of confounding | Not applicable. |
|  | Assessment of study quality, including blinding of quality assessors; stratification or regression on possible predictors of study results | The Newcastle-Ottawa Scale was applied to evaluate selection, comparability, and outcome/exposure for cohort and case-control studies. |
|  | Assessment of heterogeneity | Heterogeneity was formally tested using a chi-squared test and measured using the I^2^ statistic. |
|  | Description of statistical methods in sufficient detail to be replicated | Description of methods of meta-analyses was detailed in the data synthesis and analysis section. |
|  | Provision of appropriate tables and graphics | We included 1 flow chart and 4 summary tables. |
| **Reporting of results should include** | |  |
|  | Graph summarizing individual study estimates and overall estimate | Table 3,4 |
|  | Table giving descriptive information for each study included | Table 1,2 |
|  | Results of sensitivity testing | Effect on heterogeneity is discussed in the literature. |
|  | Indication of statistical uncertainty of findings | 95% confidence intervals were presented with all summary estimates. |
| **Reporting of discussion should include** | |  |
|  | Quantitative assessment of bias | Assessed using funnel plot and Eger’s tests (see below) |
|  | Justification for exclusion | Papers were excluded on the basis of exclusion criteria listed. We did not systematically exclude any studies on the basis of language or study population size. |
|  | Assessment of quality of included studies | The Newcastle-Ottawa Scale was used to assess the study quality. Details are showed in Table 1, 2. |
| **Reporting of conclusions should include** | |  |
|  | Consideration of alternative explanations for observed results | Only a few articles have confirmed the relationship between lifestyle habits and vitiligo. Therefore, further studies, including more high-level prospective studies and stratified studies controlling for confounding factors, are needed in order to meticulously study the effect of lifestyle habits on vitiligo. |
|  | Generalization of the conclusions | We recommend that patients with vitiligo reduce smoking and alcohol consumption; take appropriate vitamin E, B12, and zinc supplements; take vitamin C, vitamin D, and folic acid supplements as appropriate; pay attention to sun protection; and avoid permanent hair dye use. Patients with vitiligo may experience sleep disturbances and sexual dysfunction, and these patients are advised to seek help from a specialist if necessary. |
|  | Guidelines for future research | Future studies are needed to investigate the relationship between lifestyle habits and the incidence and prevalence of vitiligo. |
|  | Disclosure of funding source | Details are provided in the funding source section. |


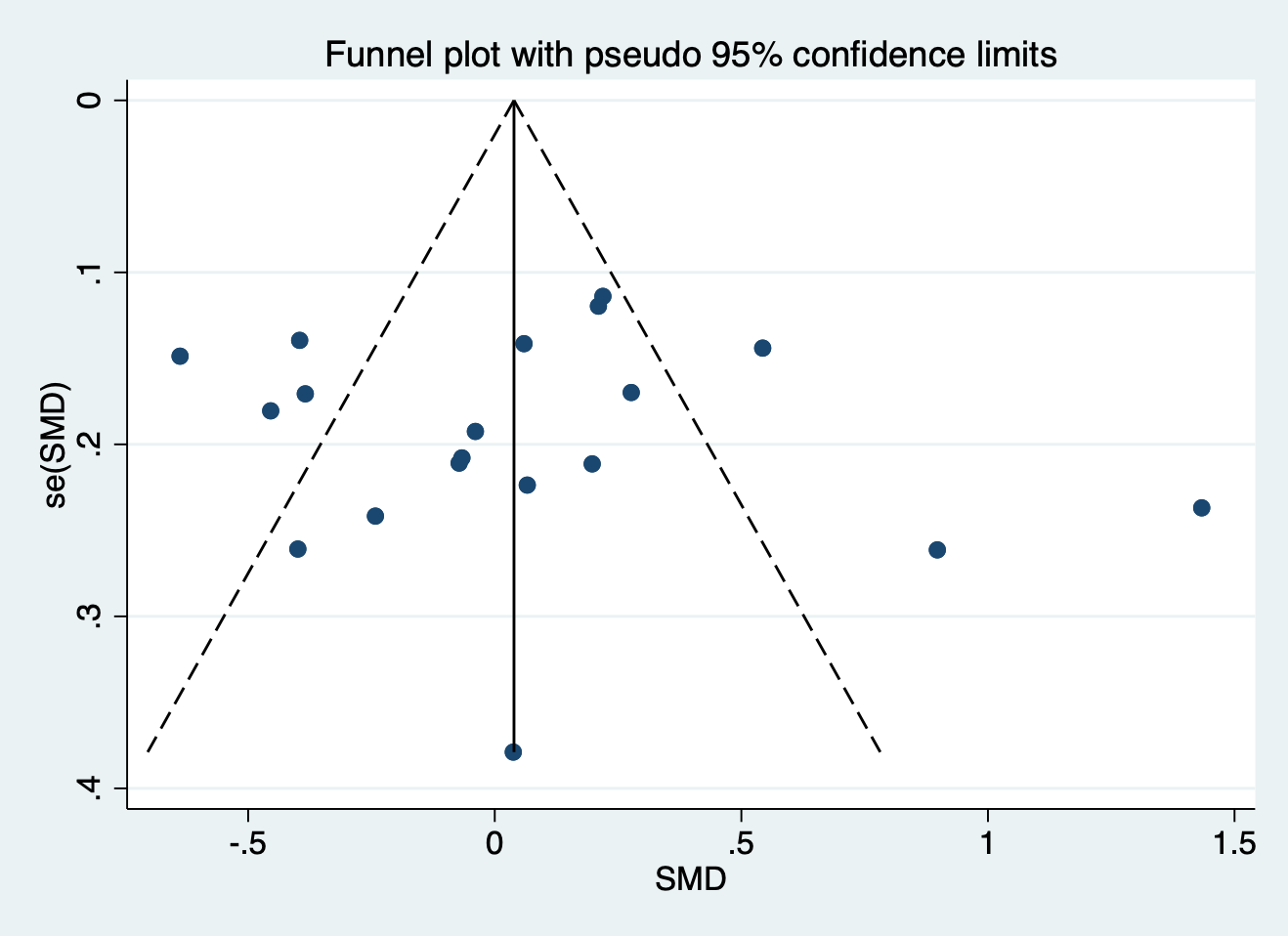


The funnel plot shows asymmetry, demonstrating that significant publication bias affects the research in this meta-analysis, and more research are needed.
