# Supplementary material for: Healthy lifestyle choices: new insights into vitiligo management
Source: Front Immunol. 2024 Nov 18;15:1440705. doi: 10.3389/fimmu.2024.1440705 (PMC11609173; doi:10.3389/fimmu.2024.1440705)
Supplement: Supplementary Table 2 — Search strategy. [file Table2.docx]

**Table S2. Search strategy for PubMed、Embase、Cochrane**

| Query | Search term |
| --- | --- |
| #1 | Vitiligo |
| #2 | Sport OR Athletics OR Athletic OR Exercises OR “Physical Activity” OR “Activities, Physical” OR “Activity, Physical” OR “Physical Activities” OR “Exercise, Physical” OR “Exercises, Physical” OR “Physical Exercise” OR “Physical Exercises” OR “Acute Exercise” OR “Acute Exercises” OR “Exercise, Acute” OR “Exercises, Acute” OR “Exercise, Isometric” OR “Exercises, Isometric” OR “Isometric Exercises”  “Isometric Exercise” OR “Exercise, Aerobic” OR “Aerobic Exercise” OR “Aerobic Exercises” OR “Exercises, Aerobic” OR “Exercise Training” OR “Exercise Trainings” OR “Training, Exercise” OR “Trainings, Exercise” |
| #3 | Smoking OR “Smoking Behaviors” OR “Behavior, Smoking” OR “Behaviors, Smoking” OR “Smoking Behavior” OR “Smoking Habit” OR “Habit, Smoking” OR “Habits, Smoking” OR “Smoking Habits” |
| #4 | “Drinking, Alcohol” OR “Alcohol Consumption” OR “Consumption, Alcohol” OR “Alcohol Intake” OR “Alcohol Intakes” OR “Intake, Alcohol” OR “Alcohol Drinking Habits” OR “Alcohol Drinking Habit” OR “Drinking Habit, Alcohol” OR “Habit, Alcohol Drinking” OR “Habits, Alcohol Drinking” |
| #5 | “Disorders of Initiating and Maintaining Sleep” OR “DIMS (Disorders of Initiating and Maintaining Sleep)” OR “Early Awakening” OR “Awakening, Early” OR “Nonorganic Insomnia” OR “Insomnia, Nonorganic” OR “Primary Insomnia” OR “Insomnia, Primary” OR “Transient Insomnia” OR “Insomnia, Transient” OR “Rebound Insomnia” OR “Insomnia, Rebound” OR “Secondary Insomnia” OR “Insomnia, Secondary” OR “Sleep Initiation Dysfunction” OR “Dysfunction, Sleep Initiation” OR “Dysfunctions, Sleep Initiation” OR  “Sleep Initiation Dysfunctions” OR Sleeplessness OR “Insomnia Disorder” OR “Insomnia Disorders” OR Insomnia OR Insomnias OR “Chronic Insomnia” OR “Insomnia, Chronic” OR “Psychophysiological Insomnia” OR “Insomnia, Psychophysiological” |
| #6 | Diets OR Diet |
| #7 | “Acid, Ascorbic” OR “L-Ascorbic Acid” OR “Acid, L-Ascorbic” OR “L Ascorbic Acid” OR Vitamin C OR Hybrin OR Magnorbin OR“Sodium Ascorbate” OR “Ascorbate, Sodium” OR “Ascorbic Acid, Monosodium Salt” OR “Ferrous Ascorbate” OR “Ascorbate, Ferrous” OR “Magnesium Ascorbate” OR “Ascorbate, Magnesium” OR “Magnesium di-L-Ascorbate” OR “Magnesium di L Ascorbate” OR “di-L-Ascorbate, Magnesium” OR “Magnesium Ascorbicum” |
| #8 | Vitamin D OR “[Receptors, Calcitriol](https://www.ncbi.nlm.nih.gov/mesh/68018167)” |
| #9 | vitamin E |
| #10 | “B 12, Vitamin” OR “Vitamin B12” OR “B12, Vitamin” OR Cyanocobalamin OR Cobalamins OR Cobalamin OR Eritron |
| #11 | “Vitamin A” OR “Aquasol A” OR Retinol OR “3,7-dimethyl-9-(2,6,6-trimethyl-1-cyclohexen-1-yl)-2,4,6,8-nonatetraen-1-ol, (all-E)-Isomer” OR “All-Trans-Retinol” OR “All Trans Retinol” OR “Vitamin A1” OR “11-cis-Retinol” |
| #12 | “Folic acid” OR “Vitamin M” OR “Vitamin B9” OR “B9, Vitamin” OR “Pteroylglutamic Acid” OR “Folic Acid, Monopotassium Salt” OR “Folic Acid, Monosodium Salt” OR “Folic Acid, Potassium Salt” OR “Folic Acid, (DL)-Isomer” OR Folvite OR Folacin OR Folate OR “Folic Acid, (D)-Isomer” OR “Folic Acid, Calcium Salt (1:1)” OR “Folic Acid, Sodium Salt” |
| #13 | Zinc |
| #14 | Copper OR “Copper-63” OR “Copper 63” |
| #15 | Selenium OR “Selenium-80” OR “Selenium 80” |
| #16 | Iron OR “Iron-56” OR “Iron 56” |
| #17 | Sunshine OR Sunlight |
| #18 | Height OR “Body Heights” OR “Height, Body” OR “Heights, Body” |
| #19 | Permanent hair dyes |
| #20 | Sleeping OR DIMS (Disorders of Initiating and Maintaining Sleep) OR “Disorders of Initiating and Maintaining Sleep” OR Sleeplessness OR “Insomnia Disorder” OR “Insomnia Disorders” OR Insomnia OR Insomnias OR “Chronic Insomnia” OR “Insomnia, Chronic” OR “Early Awakening” OR “Awakening, Early” OR “Nonorganic Insomnia” OR “Insomnia, Nonorganic” OR “Primary Insomnia” OR “Insomnia, Primary” OR “Psychophysiological Insomnia” OR “Insomnia, Psychophysiological” OR “Rebound Insomnia” OR “Insomnia, Rebound” OR “Secondary Insomnia” OR “Insomnia, Secondary” OR “Sleep Initiation Dysfunction” OR “Dysfunction, Sleep Initiation” OR “Dysfunctions, Sleep Initiation” OR “Sleep Initiation Dysfunctions” OR “Transient Insomnia” OR “Insomnia, Transient” |
| #21 | Tanning ability |
| #22 | #1 AND (#2 OR #3 OR #4 OR #5 OR #6 OR #7 OR #8 OR #9 OR #10 OR #11 OR #12 OR #13 OR #14 OR #15 OR #16 OR #17 OR #18 OR #19 OR #20 OR #21) |
